# Crystal structures of three 1-oxo-1,2-di­hydro­naphthalene derivatives: dimethyl 4-(4-meth­oxy­phen­yl)-2-(4-methyl­phen­yl)-1-oxo-1,2-di­hydro­naphthalene-2,3-di­carboxyl­ate, dimethyl 1-oxo-2-(pyren-4-yl)-4-(thio­phen-2-yl)-1,2-di­hydro­naphthalene-2,3-di­carboxyl­ate and ethyl 1-oxo-2-phenyl-2,4-bis­(thio­phen-2-yl)-1,2-di­hydro­naphthalene-3-carboxyl­ate

**DOI:** 10.1107/S2056989017000469

**Published:** 2017-01-13

**Authors:** S. Gopinath, P. Narayanan, K. Sethusankar, Jeyachandran Karunakaran, Meganathan Nandakumar, Arasambattu K. Mohanakrishnan

**Affiliations:** a Department of Physics, RKM Vivekananda College (Autonomous), Chennai 600 004, India; b Department of Organic Chemistry, University of Madras, Guindy Campus, Chennai 600 025, India

**Keywords:** crystal structure, 1-oxo-1,2-di­hydro­naphthalene, pyrene, thio­phene, hydrogen bonding

## Abstract

In the title 1-oxo-1,2-di­hydro­naphthalene derivatives, C_28_H_24_O_6_, (I), C_34_H_22_O_5_S, (II), and C_27_H_20_O_3_S_2_, (III), the cyclo­hexa-1,3-diene rings of the 1,2-di­hydro­naphthalene ring systems adopt half-chair, boat and half-chair conformations, respectively. The carbonyl O atoms attached to the di­hydro­naphthalene ring systems are each significantly deviated from the mean plane of the 1,2-di­hydro­naphthalene ring system, by 0.6162 (12) Å in (I), 0.6016 (16) Å in (II) and 0.515 (3) Å in (III). The mean planes of the 1,2-di­hydro­naphthalene ring systems make dihedral angles of 85.83 (3), 88.19 (3) and 81.67 (8)°, respectively, with the methyl­phenyl ring in (I), the pyrene ring in (II) and the phenyl ring in (III). In (I), the mol­ecular structure is stabilized by an intra­molecular C—H⋯O hydrogen bond, generating an *S*(6) ring motif. In the crystal of (I), mol­ecules are linked by an inter­molecular C—H⋯O hydrogen bond, which generates a *C*(8) zigzag chain running along [100]. Adjacent chains are further connected by C—H⋯π and offset π–π inter­actions [centroid–centroid distance = 3.6572 (9) Å], forming a double-chain structure. In the crystals of (II) and (III), mol­ecules are linked into chain structures by offset π–π inter­actions with centroid–centroid distances of 3.5349 (12) and 3.8845 (13) Å for (II) and 3.588 (2) Å for (III). In (II) and (III), the thio­phene rings are orientationally disordered over two sites, with occupancy ratios of 0.69:0.31 for (II), and 0.528 (4):0.472 (4) and 0.632 (5):0.368 (5) for (III).

## Abstract 2

In the title 1-oxo-1,2-di­hydro­naphthalene derivatives, the cyclo­hexa-1,3-diene rings of the 1,2-di­hydro­naphthalene ring systems adopt half-chair, boat and half-chair conformations, respectively. In the crystal of the methyl­phenyl compound, the mol­ecules are linked *via* C—H⋯O, C—H⋯π and π–π inter­actions, forming a double-chain structure, while in the crystals of the other two compounds, mol­ecules are linked by π–π inter­actions, forming a chain structure.

## Chemical context   

Naphthalene derivatives have been employed extensively in many fields, and some of them possess important biological and commercial applications, including use as disinfectants, insecticides and auxin plant hormones, and rooting agents (Morikawa & Takahashi, 2004 ▸). The bicyclic naphthalene skeleton constitutes a large number of clinical drugs, such as propranolol (Crowther & Smith, 1968 ▸), naproxen (Harrison *et al.*, 1970 ▸), an anti-inflammatory agent (Goudie *et al.*, 1978 ▸) and methallenestril (a non-steroid oestrogen). Di­hydroxy­naph­thal­ene derivatives are a class of inter­mediates important for applications in dye synthesis (Bianchi *et al.*, 1997 ▸) or as monomers in the preparation of polymers, such as polyesters (Blundell & Buckingham, 1985 ▸; Aitken *et al.*, 1992 ▸) and polynapthooxazines (Shen & Ishida, 1996 ▸). 1,2,3,4-Tetra­hydro­naphthalene derivatives are used for the treatment of central nervous system disorders (Jerussi *et al.*, 2004 ▸; Taber *et al.*, 2004 ▸). Tetra­hydro­naphthalene derivatives are also used in liquid crystal display elements (Ray *et al.*, 2003 ▸). 1-Naphthalene­acetic acid is well known as a growth regulator/stimulator in a variety of fruits and vegetables (Garriz *et al.*, 2004 ▸; Li *et al.*, 2004 ▸). Against this background, we synthesized the title compounds (I), (II) and (III) and report herein on their crystal structures and mol­ecular conformations.
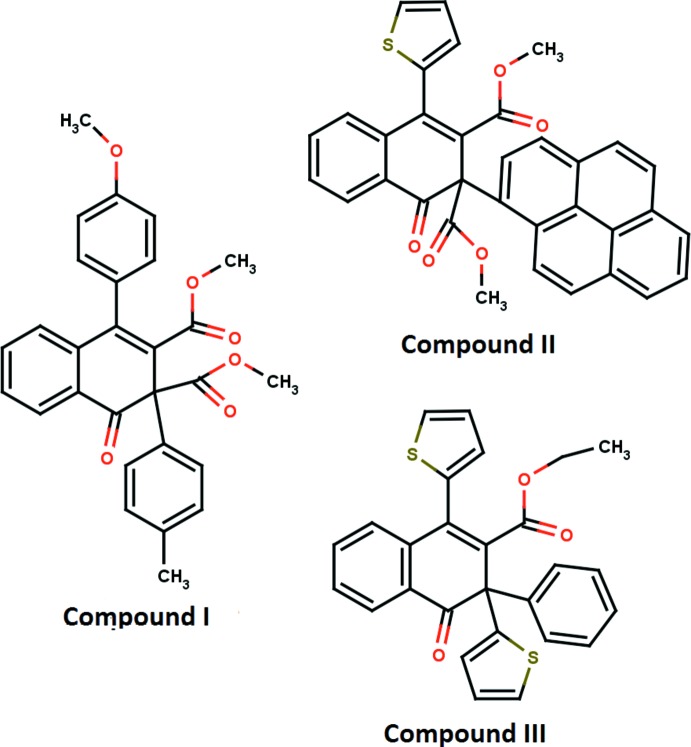


## Structural commentary   

**Figure 1:**
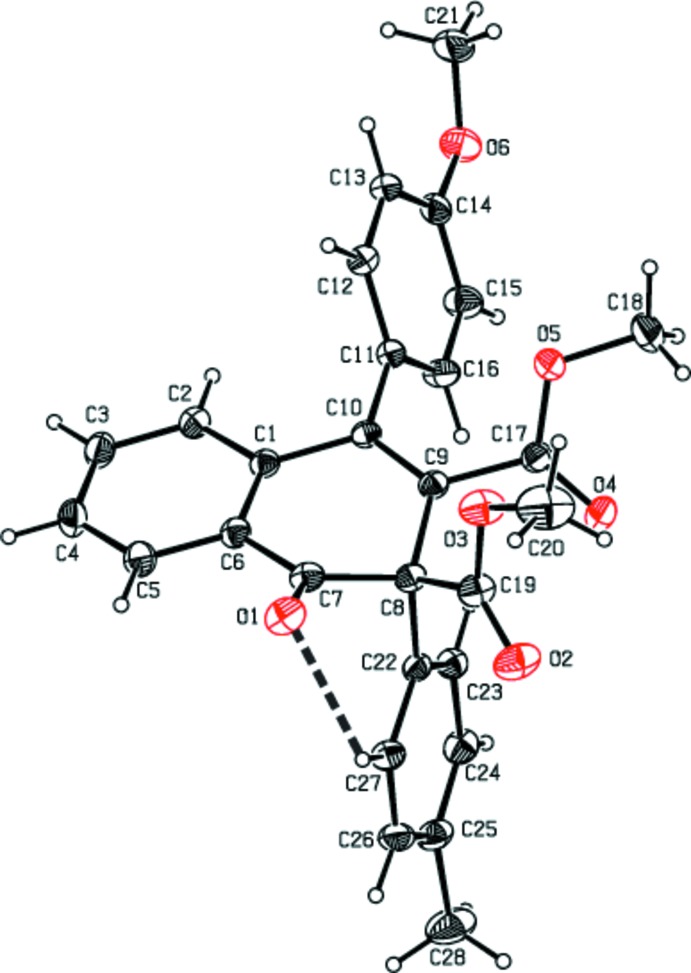
The mol­ecular structure of compound (I), with the atom-numbering scheme. The intra­molecular C—H⋯O inter­action with an *S*(6) ring motif is shown as a dashed line. Displacement ellipsoids are drawn at the 30% probability level. H atoms are shown as spheres of arbitrary radius.

The mol­ecular structures of the title compounds (I), (II) and (III) are shown in Figs. 1 ▸, 2 ▸ and 3 ▸, respectively. The cyclo­hexa-1,3-diene rings (C1/C6–C10) of the 1,2-di­hydro naphthalene ring systems of compounds (I), (II) and (III) adopt half-chair, boat and half-chair conformations, respectively, with puckering and smallest displacement parameters of *q* = 0.3370 (16) Å, *θ* = 115.7 (3)°, *φ* = 337.2 (3)° and ΔC_s_ = 5.4 (2) for (I), *q* = 0.257 (2) Å, *θ* = 66.6 (4)°, *φ* = 136.9 (5)° and ΔC_s_ = 6.9 (2) for (II), and *q* = 0.287 (3) Å, *θ* = 114.7 (6)°, *φ* = 337.2 (7)° and ΔC_s_ = 4.4 (4) for (III). In each compound, the carbonyl oxygen atom O1 deviates significantly from the mean plane of the 1,2-di­hydro­napthalene ring system [by 0.6453 (13) Å for (I), 0.6016 (16) Å for (II) and 0.548 (3) Å for (III)]. The mean planes of the 1,2-di­hydro­naphthalene ring systems make dihedral angles of 85.83 (3), 88.19 (3) and 81.67 (8)° with the methyl­phenyl ring in (I), the pyrene ring in (II) and the phenyl ring in (III).

In (I), the meth­oxy­phenyl ring is inclined by 19.41 (5) and 67.84 (4)°, respectively, to the methyl­phenyl ring and the mean plane of 1,2-di­hydro­naphthalene ring system. The methyl group carbon atom C28 deviates slightly [by 0.115 (2) Å] from the C22–C27 ring. The mol­ecular structure is stabilized by an intra­molecular C—H⋯O hydrogen bond with an *S*(6) ring motif (Table 1 ▸). In (II), the pyrene moiety is essentially planar with a maximum deviation of 0.085 (2) Å for atom C27. The thio­phene ring is orientationally disordered over two sites with an occupancy ratio of 0.69:0.31. In (III), the two thio­phene rings are also disordered with occupancy ratios of 0.528 (4):0.472 (4) and 0.632 (5):0.368 (5).

## Supra­molecular features   

**Figure 4:**
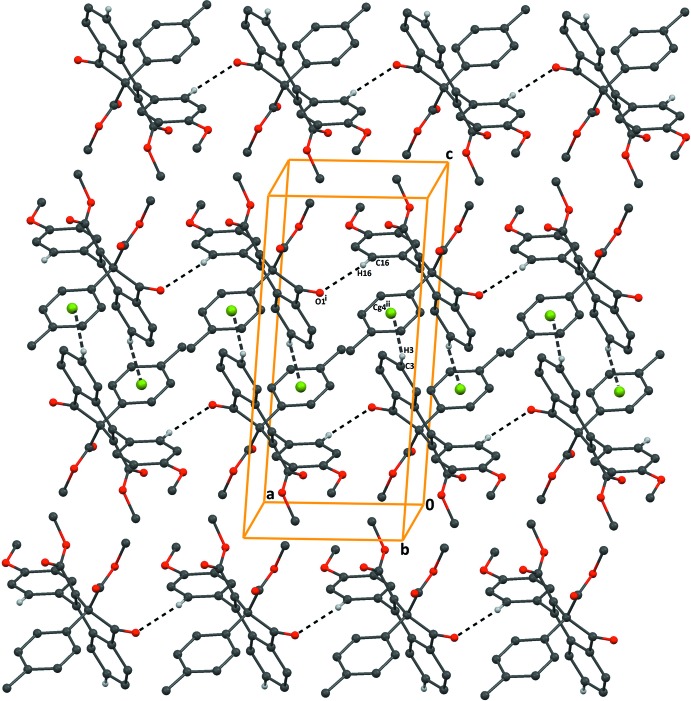
A packing diagram of compound (I), viewed along the *b* axis, showing the C16—H16⋯O1^i^ and C3—H3⋯*Cg*4^ii^ inter­actions (dashed lines). *Cg*4 is the centroid of the C22–C27 benzene ring. [Symmetry codes: (i) −1 + *x*, *y*, *z*; (ii) 2 − *x*, 1 − *y*, 1 − *z*.]

In the crystal of compound (I), the mol­ecules are linked *via* C—H⋯O hydrogen bonds (C16—H16⋯O1^i^; Table 1 ▸), generating a *C*(8) zigzag chain along to [100]. Adjacent chains are further linked into a double-chain structure (Fig. 4 ▸) through C—H⋯π and π–π inter­actions [C3—H3⋯*Cg*4^ii^; Table 1 ▸; *Cg*1⋯*Cg*1^ii^ = 3.6572 (9) Å, inter­planar distance = 3.443 (1) Å, slippage = 1.232 Å; *Cg*1 and *Cg*4 are the centroids of the C1–C6 and C22–C27 benzene rings, respectively].

**Figure 5:**
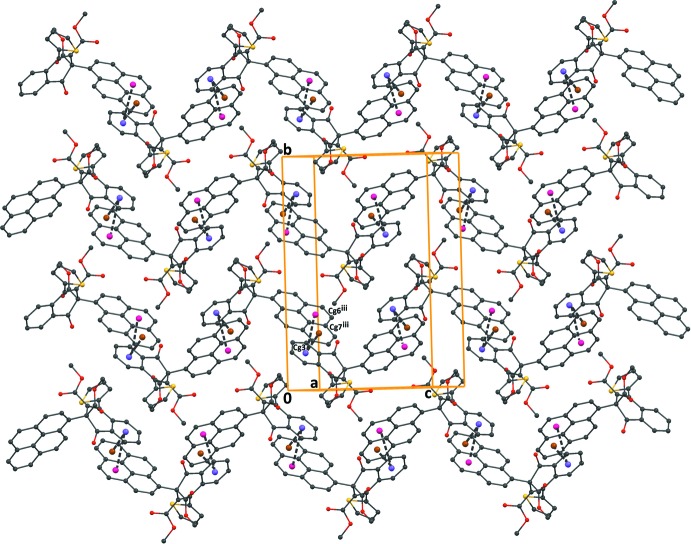
A packing diagram of compound (II), viewed approximately along the *a* axis, showing the π–π inter­actions (dashed lines). H atoms have been omitted for clarity. *Cg*3, *Cg*6 and *Cg*7 are the centroids of the C1–C6, C22–C25/C33/C34 and C25–C29/C34 benzene rings, respectively. [Symmetry code: (iii) −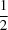 + *x*, 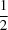 − *y*, −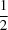 + *z*.]

**Figure 6:**
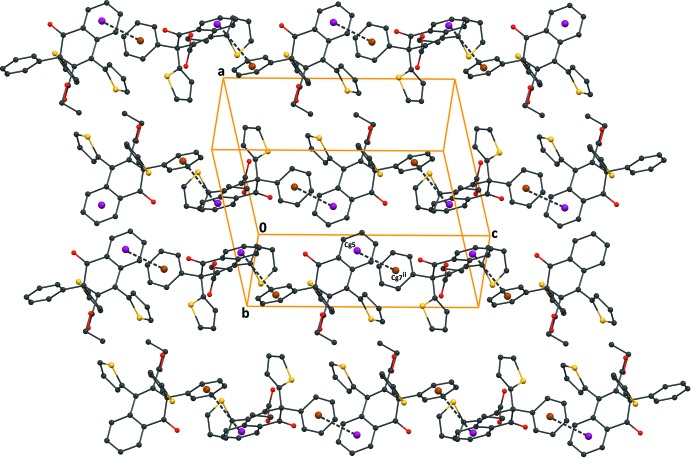
A packing diagram of compound (III), showing the π–π inter­actions (dashed lines). H atoms have been excluded for clarity. *Cg*5 and *Cg*7 are the centroids of the C1–C6 and C22–C27 benzene rings, respectively. [Symmetry code: (iv) *x*, 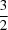 − *y*, −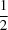 + *z*.]

In the crystal of (II), the mol­ecules are linked by offset π–π inter­actions, forming a chain along [101] [*Cg*3⋯*Cg*6^iii^ = 3.5349 (12) Å, inter­planar distance = 3.466 (1) Å; *Cg*3⋯*Cg*7^iii^ = 3.8845 (13) Å, inter­planar distance = 3.468 (1) Å; *Cg*3, *Cg*6 and *Cg*7 are the centroids of the C1–C6, C22–C25/C33/C34 and C25–C29/C34 benzene rings, respectively; symmetry code: (iii) −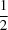 + *x*, 1/2-*y*, −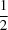 + *z*; Fig. 5 ▸]. In the crystal of (III), the mol­ecules are linked into a chain along [001] by an offset π–π inter­action [*Cg*5⋯*Cg*7^iv^ = 3.888 (2) Å, inter­planar distance = 3.632 (1) Å; *Cg*5 and *Cg*7 are the centroids of the benzene C1–C6 and C22–C27 rings, respectively; symmetry code: (iv) *x*, 3/2-*y*, 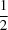 + *z*; Fig. 6 ▸].

## Synthesis and crystallization   

**Compound (I)**: To a stirred solution of 1-(4-meth­oxy­phen­yl)-3-*p*-tolyl­isobenzo­furan (1 g, 3.31 mmol) in dry di­chloro­methane (DCM), dimethyl acetyl­enedi­carboxyl­ate (DMAD) (0.52 g, 3.64 mmol) was added and the reaction mixture was stirred at room temperature for 1 h. Removal of the solvent was followed by column chromatographic purification (silica gel; 15% ethyl acetate in hexa­ne) gave the isobenzo­furan–DMAD adduct as a colorless solid (1.31 g, 87%). To a stirred solution of isobenzo­furan–DMAD adduct (0.30 g, 0.678 mmol) in dry DCM, BF_3_·OEt_2_ (0.04 g, 0.28 mmol) was added and the reaction mixture was stirred at room temperature for 5 min. Removal of the solvent followed by column chromatographic purification (silica gel; 15% ethyl acetate in hexa­ne) gave compound (I) (0.28 g, 94%) as a colorless solid. Single crystals suitable for X-ray diffraction were prepared by slow evaporation from an ethyl acetate solution of (I) at room temperature, m.p. 480–481 K.

**Compound (II)**: To a stirred solution of 1-(pyren-1-yl)-3-(thio­phen-2-yl)isobenzo­furan (0.50 g, 1.25 mmol) in dry DCM (10 ml), DMAD (0.19 g, 1.32 mmol) was added and the reaction mixture was stirred at room temperature for 1 h. To this, BF_3_·OEt_2_ (0.075 g, 0.53 mmol) was added and stirred at room temperature for 5 min. Removal of the solvent followed by column chromatographic purification (silia gel; 15% ethyl acetate in hexa­ne) afforded compound (II) as a yellow solid. Single crystals suitable for X-ray diffraction were prepared by slow evaporation from an ethyl acetate solution of (II) at room temperature, m.p. 469–471 K.

**Compound (III)**: To a solution of 1,3-di(thio­phen-2-yl)isobenzo furan (0.50 g, 1.77 mmol) in dry toluene (15 ml), ethyl-3-phenyl­propiolate (0.34 g, 1.95 mmol) was added and refluxed till the consumption of 1,3-di(thio­phen-2-yl)isobenzo­furan (disappearance of fluorescent colour in 8 h). After removal of toluene *in vacuo*, the crude adduct was dissolved in dry DCM (15 ml), BF_3_·OEt_2_ (0.075 g, 0.52 mmol) was added and the reaction mixture was stirred for 10 min at room temperature. Removal of the solvent was followed by column chromatographic purification (silica gel; 15% ethyl accetate in hexa­ne) which afforded compound (III) as a green solid (0.53 g, 65%). Single crystals suitable for X-ray diffraction were prepared by slow evaporation from an ethyl acetate solution of (III) at room temperature, m.p. 383–385 K.

## Refinement   

Crystal data, data collection and structure refinement details are summarized in Table 2 ▸. For all compounds, H atoms were localized in difference Fourier maps and were then constrained geometrically with C—H = 0.93, 0.96 and 0.97 Å for aryl, methyl and methyl­ene H atoms, respectively, allowing for rotation of the methyl groups. The *U*_iso_(H) values were set to 1.5*U*_eq_(C) for methyl H atoms and 1.2*U*_eq_(C) for other H atoms. In compound (II), the thio­phene ring is disordered and the occupancy ratio was refined to 0.691 (3):0.309 (3), which was then fixed at 0.69:0.31 in the final refinement. In compound (III), the two thio­phene rings are disordered with refined occupancy ratios of 0.528 (4):0.472 (4) and 0.632 (5):0.368 (5). For (II) and (III), ellipsoid displacement restraints (*SIMU* and *DELU*) and bond length restraints (*DFIX*) with C—S = 1.70 (1) Å, C—C = 1.50 (1) Å and C=C = 1.40 (1) Å were applied to the disordered rings.

## Supplementary Material

Crystal structure: contains datablock(s) I, II, III, global. DOI: 10.1107/S2056989017000469/is5470sup1.cif

Structure factors: contains datablock(s) I. DOI: 10.1107/S2056989017000469/is5470Isup2.hkl

Structure factors: contains datablock(s) II. DOI: 10.1107/S2056989017000469/is5470IIsup3.hkl

Structure factors: contains datablock(s) III. DOI: 10.1107/S2056989017000469/is5470IIIsup4.hkl

Supporting information file. DOI: 10.1107/S2056989017000469/is5470Isup5.cml

Supporting information file. DOI: 10.1107/S2056989017000469/is5470IIsup6.cml

Supporting information file. DOI: 10.1107/S2056989017000469/is5470IIIsup7.cml

CCDC references: 997379, 1438209, 1438503

Additional supporting information:  crystallographic information; 3D view; checkCIF report

## Figures and Tables

**Figure 2:**
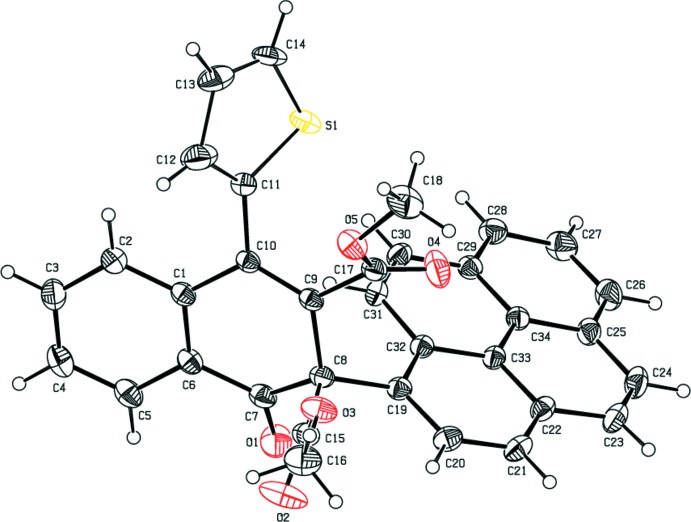
The mol­ecular structure of compound (II), with the atom-numbering scheme. Displacement ellipsoids are drawn at the 30% probability level. H atoms are shown as spheres of arbitrary radius. For the sake of clarity, the minor component of the disordered thio­phene ring has been omitted.

**Figure 3:**
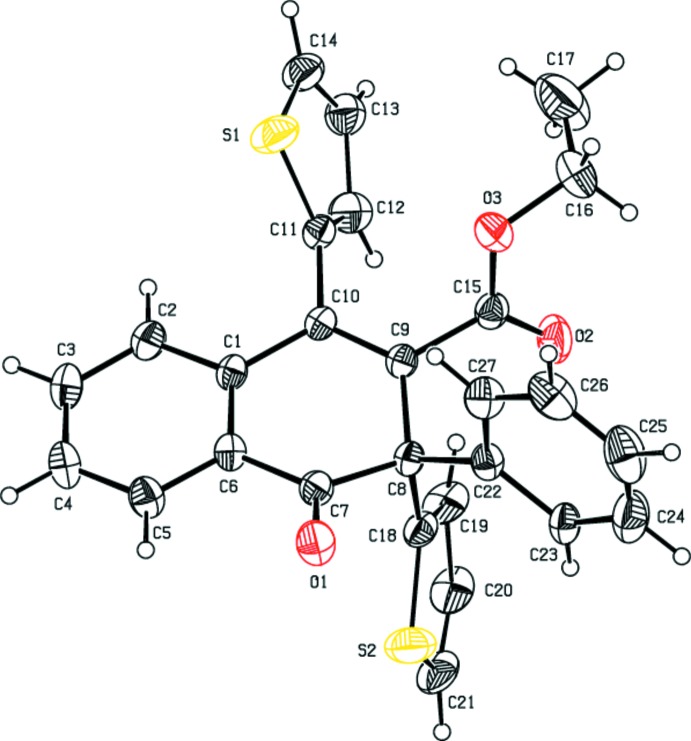
The mol­ecular structure of compound (III), with the atom-numbering scheme. Displacement ellipsoids are drawn at the 30% probability level. H atoms are shown as spheres of arbitrary radius. For the sake of clarity, the minor components of the disordered thio­phene rings have been omitted.

**Table 1:** Hydrogen-bond geometry (Å, °) for (I) *Cg*4 is the centroid of the C22–C27 benzene ring.

*D*—H⋯*A*	*D*—H	H⋯*A*	*D*⋯*A*	*D*—H⋯*A*
C27—H27⋯O1	0.93	2.52	3.109 (2)	121
C16—H16⋯O1^i^	0.93	2.52	3.344 (3)	148
C3—H3⋯*Cg*4^ii^	0.93	2.78	3.656 (2)	157

Symmetry codes: (i) 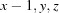; (ii) 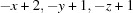.

**Table 2:** Experimental details

	(I)	(II)	(III)
Crystal data			
Chemical formula	C_28_H_24_O_6_	C_34_H_22_O_5_S	C_27_H_20_O_3_S_2_
*M*_r_	456.47	542.58	456.55
Crystal system, space group	Triclinic, *P* 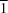	Monoclinic, *P*2_1_/*n*	Monoclinic, *P*2_1_/*c*
Temperature (K)	296	296	296
*a*, *b*, *c* (Å)	7.5256 (2), 10.2095 (3), 15.6299 (4)	10.9268 (10), 18.9670 (14), 12.2628 (9)	12.1263 (11), 11.8009 (11), 16.0657 (13)
α, β, γ (°)	93.990 (1), 94.679 (1), 101.089 (2)	90, 93.030 (2), 90	90, 100.181 (2), 90
*V* (Å^3^)	1170.06 (6)	2537.9 (4)	2262.8 (3)
*Z*	2	4	4
Radiation type	Mo *K*α	Mo *K*α	Mo *K*α
μ (mm^−1^)	0.09	0.17	0.26
Crystal size (mm)	0.35 × 0.30 × 0.25	0.25 × 0.25 × 0.20	0.25 × 0.25 × 0.15

Data collection			
Diffractometer	Bruker Kappa APEXII CCD	Bruker Kappa APEXII CCD	Bruker Kappa APEXII CCD
Absorption correction	Multi-scan (*SADABS*; Bruker, 2008 ▸)	Multi-scan (*SADABS*; Bruker, 2008 ▸)	Multi-scan (*SADABS*; Bruker, 2008 ▸)
*T*_min_, *T*_max_	0.969, 0.978	0.958, 0.966	0.937, 0.962
No. of measured, independent and observed [*I* > 2σ(*I*)] reflections	22413, 4119, 3346	21576, 4457, 3341	29901, 4110, 2685
*R*_int_	0.027	0.031	0.044
(sin θ/λ)_max_ (Å^−1^)	0.595	0.595	0.603

Refinement			
*R*[*F* ^2^ > 2σ(*F* ^2^)], *wR*(*F* ^2^), *S*	0.040, 0.111, 1.03	0.039, 0.105, 1.04	0.061, 0.147, 1.09
No. of reflections	4119	4457	4110
No. of parameters	311	400	364
No. of restraints	0	56	100
H-atom treatment	H-atom parameters constrained	H-atom parameters constrained	H-atom parameters constrained
Δρ_max_, Δρ_min_ (e Å^−3^)	0.19, −0.21	0.22, −0.22	0.24, −0.24

Computer programs: *APEX2*and *SAINT* (Bruker, 2008 ▸), *SHELXS97* and *SHELXL97* (Sheldrick, 2008 ▸), *ORTEP-3 for Windows* (Farrugia, 2012 ▸), *Mercury* (Macrae *et al.*, 2008 ▸) and *PLATON* (Spek, 2015 ▸).

## supplementary crystallographic information

### (I) Dimethyl 4-(4-methoxyphenyl)-2-(4-methylphenyl)-1-oxo-1,2-dihydronaphthalene-2,3-dicarboxylate Crystal data

**Table:**

C_28_H_24_O_6_	*Z* = 2
*M**_r_* = 456.47	*F*(000) = 480
Triclinic, *P*1	*D*_x_ = 1.296 Mg m^−^^3^
Hall symbol: -P 1	Mo *K*α radiation, λ = 0.71073 Å
*a* = 7.5256 (2) Å	Cell parameters from 4119 reflections
*b* = 10.2095 (3) Å	θ = 2.3–25.0°
*c* = 15.6299 (4) Å	µ = 0.09 mm^−^^1^
α = 93.990 (1)°	*T* = 296 K
β = 94.679 (1)°	Block, colourless
γ = 101.089 (2)°	0.35 × 0.30 × 0.25 mm
*V* = 1170.06 (6) Å^3^	

### (I) Dimethyl 4-(4-methoxyphenyl)-2-(4-methylphenyl)-1-oxo-1,2-dihydronaphthalene-2,3-dicarboxylate Data collection

**Table:**

Bruker Kappa APEXII CCD diffractometer	4119 independent reflections
Radiation source: fine-focus sealed tube	3346 reflections with *I* > 2σ(*I*)
Graphite monochromator	*R*_int_ = 0.027
ω &φ scans	θ_max_ = 25.0°, θ_min_ = 2.3°
Absorption correction: multi-scan (SADABS; Bruker, 2008)	*h* = −8→8
*T*_min_ = 0.969, *T*_max_ = 0.978	*k* = −12→12
22413 measured reflections	*l* = −18→18

### (I) Dimethyl 4-(4-methoxyphenyl)-2-(4-methylphenyl)-1-oxo-1,2-dihydronaphthalene-2,3-dicarboxylate Refinement

**Table:**

Refinement on *F*^2^	Primary atom site location: structure-invariant direct methods
Least-squares matrix: full	Secondary atom site location: difference Fourier map
*R*[*F*^2^ > 2σ(*F*^2^)] = 0.040	Hydrogen site location: inferred from neighbouring sites
*wR*(*F*^2^) = 0.111	H-atom parameters constrained
*S* = 1.03	*w* = 1/[σ^2^(*F*_o_^2^) + (0.0547*P*)^2^ + 0.2906*P*] where *P* = (*F*_o_^2^ + 2*F*_c_^2^)/3
4119 reflections	(Δ/σ)_max_ = 0.004
311 parameters	Δρ_max_ = 0.19 e Å^−^^3^
0 restraints	Δρ_min_ = −0.21 e Å^−^^3^

### (I) Dimethyl 4-(4-methoxyphenyl)-2-(4-methylphenyl)-1-oxo-1,2-dihydronaphthalene-2,3-dicarboxylate Special details

**Table:**

Geometry. All esds (except the esd in the dihedral angle between two l.s. planes) are estimated using the full covariance matrix. The cell esds are taken into account individually in the estimation of esds in distances, angles and torsion angles; correlations between esds in cell parameters are only used when they are defined by crystal symmetry. An approximate (isotropic) treatment of cell esds is used for estimating esds involving l.s. planes.
Refinement. Refinement of F^2^ against ALL reflections. The weighted R-factor wR and goodness of fit S are based on F^2^, conventional R-factors R are based on F, with F set to zero for negative F^2^. The threshold expression of F^2^ > 2sigma(F^2^) is used only for calculating R-factors(gt) etc. and is not relevant to the choice of reflections for refinement. R-factors based on F^2^ are statistically about twice as large as those based on F, and R- factors based on ALL data will be even larger.

### (I) Dimethyl 4-(4-methoxyphenyl)-2-(4-methylphenyl)-1-oxo-1,2-dihydronaphthalene-2,3-dicarboxylate Fractional atomic coordinates and isotropic or equivalent isotropic displacement parameters (Å^2^)

**Table:**

	*x*	*y*	*z*	*U*_iso_*/*U*_eq_	
C1	1.04016 (19)	0.52685 (14)	0.34053 (9)	0.0385 (3)	
C2	1.0229 (2)	0.63513 (15)	0.39596 (10)	0.0478 (4)	
H2	0.9373	0.6863	0.3807	0.057*	
C3	1.1303 (2)	0.66814 (17)	0.47312 (11)	0.0550 (4)	
H3	1.1160	0.7407	0.5095	0.066*	
C4	1.2578 (2)	0.59471 (18)	0.49645 (11)	0.0587 (5)	
H4	1.3297	0.6170	0.5486	0.070*	
C5	1.2791 (2)	0.48799 (18)	0.44251 (11)	0.0550 (4)	
H5	1.3667	0.4387	0.4581	0.066*	
C6	1.17134 (19)	0.45323 (14)	0.36521 (10)	0.0417 (3)	
C7	1.1958 (2)	0.33854 (15)	0.30791 (10)	0.0446 (4)	
C8	1.0303 (2)	0.27105 (14)	0.24589 (9)	0.0397 (3)	
C9	0.92937 (19)	0.37508 (14)	0.21154 (9)	0.0385 (3)	
C10	0.92853 (19)	0.49101 (14)	0.25739 (9)	0.0371 (3)	
C11	0.8042 (2)	0.58099 (14)	0.23090 (9)	0.0401 (3)	
C12	0.8649 (2)	0.70937 (15)	0.20884 (10)	0.0479 (4)	
H12	0.9892	0.7416	0.2088	0.058*	
C13	0.7446 (3)	0.79111 (15)	0.18672 (10)	0.0521 (4)	
H13	0.7878	0.8768	0.1710	0.063*	
C14	0.5606 (2)	0.74492 (16)	0.18809 (10)	0.0509 (4)	
C15	0.4985 (2)	0.61764 (18)	0.21056 (12)	0.0594 (5)	
H15	0.3743	0.5861	0.2118	0.071*	
C16	0.6190 (2)	0.53671 (16)	0.23124 (11)	0.0522 (4)	
H16	0.5749	0.4504	0.2458	0.063*	
C17	0.8254 (2)	0.33536 (15)	0.12560 (10)	0.0457 (4)	
C18	0.7520 (3)	0.4012 (2)	−0.01137 (12)	0.0833 (7)	
H18A	0.7824	0.3208	−0.0363	0.125*	
H18B	0.7902	0.4735	−0.0461	0.125*	
H18C	0.6228	0.3879	−0.0088	0.125*	
C19	1.0997 (2)	0.19417 (17)	0.17186 (11)	0.0523 (4)	
C20	1.2394 (4)	0.2216 (3)	0.04350 (16)	0.1075 (10)	
H20A	1.3518	0.1943	0.0597	0.161*	
H20B	1.2588	0.2863	0.0018	0.161*	
H20C	1.1505	0.1449	0.0190	0.161*	
C21	0.4758 (4)	0.9350 (2)	0.12847 (14)	0.0822 (7)	
H21A	0.5676	0.9976	0.1647	0.123*	
H21B	0.3702	0.9736	0.1181	0.123*	
H21C	0.5219	0.9148	0.0746	0.123*	
C22	0.9152 (2)	0.17131 (13)	0.29922 (9)	0.0383 (3)	
C23	0.7324 (2)	0.16747 (15)	0.30518 (9)	0.0435 (4)	
H23	0.6760	0.2297	0.2789	0.052*	
C24	0.6325 (2)	0.07153 (17)	0.35007 (11)	0.0536 (4)	
H24	0.5091	0.0698	0.3528	0.064*	
C25	0.7110 (3)	−0.02137 (16)	0.39079 (11)	0.0569 (5)	
C26	0.8952 (3)	−0.01318 (16)	0.38729 (11)	0.0575 (5)	
H26	0.9528	−0.0724	0.4162	0.069*	
C27	0.9956 (2)	0.08046 (15)	0.34213 (10)	0.0494 (4)	
H27	1.1194	0.0828	0.3404	0.059*	
C28	0.6003 (4)	−0.1312 (2)	0.43490 (16)	0.0927 (8)	
H28A	0.4822	−0.1116	0.4415	0.139*	
H28B	0.6608	−0.1372	0.4906	0.139*	
H28C	0.5874	−0.2150	0.4007	0.139*	
O1	1.33429 (15)	0.29405 (12)	0.31246 (9)	0.0629 (3)	
O2	1.0894 (2)	0.07656 (13)	0.16239 (9)	0.0765 (4)	
O3	1.17487 (19)	0.28042 (13)	0.11870 (8)	0.0680 (4)	
O4	0.7426 (2)	0.22389 (12)	0.10391 (8)	0.0720 (4)	
O5	0.84266 (17)	0.43348 (11)	0.07425 (7)	0.0588 (3)	
O6	0.42858 (19)	0.81619 (13)	0.16963 (9)	0.0736 (4)	

### (I) Dimethyl 4-(4-methoxyphenyl)-2-(4-methylphenyl)-1-oxo-1,2-dihydronaphthalene-2,3-dicarboxylate Atomic displacement parameters (Å^2^)

**Table:**

	*U*^11^	*U*^22^	*U*^33^	*U*^12^	*U*^13^	*U*^23^
C1	0.0357 (8)	0.0370 (7)	0.0416 (8)	0.0024 (6)	0.0060 (6)	0.0053 (6)
C2	0.0525 (9)	0.0418 (8)	0.0484 (9)	0.0102 (7)	0.0033 (7)	0.0000 (7)
C3	0.0607 (11)	0.0499 (9)	0.0493 (9)	0.0035 (8)	0.0019 (8)	−0.0061 (7)
C4	0.0537 (10)	0.0613 (11)	0.0525 (10)	−0.0007 (8)	−0.0102 (8)	−0.0034 (8)
C5	0.0391 (9)	0.0574 (10)	0.0646 (11)	0.0051 (7)	−0.0072 (8)	0.0061 (8)
C6	0.0318 (8)	0.0415 (8)	0.0495 (8)	0.0014 (6)	0.0035 (6)	0.0044 (6)
C7	0.0355 (8)	0.0419 (8)	0.0577 (9)	0.0070 (7)	0.0095 (7)	0.0103 (7)
C8	0.0398 (8)	0.0357 (7)	0.0450 (8)	0.0086 (6)	0.0099 (6)	0.0031 (6)
C9	0.0377 (8)	0.0368 (7)	0.0407 (7)	0.0038 (6)	0.0075 (6)	0.0057 (6)
C10	0.0359 (8)	0.0346 (7)	0.0404 (7)	0.0035 (6)	0.0073 (6)	0.0064 (6)
C11	0.0462 (9)	0.0358 (7)	0.0380 (7)	0.0076 (6)	0.0027 (6)	0.0035 (6)
C12	0.0503 (9)	0.0400 (8)	0.0504 (9)	0.0023 (7)	0.0011 (7)	0.0060 (7)
C13	0.0724 (12)	0.0339 (8)	0.0481 (9)	0.0083 (8)	−0.0030 (8)	0.0071 (7)
C14	0.0622 (11)	0.0471 (9)	0.0459 (8)	0.0219 (8)	−0.0059 (7)	0.0024 (7)
C15	0.0471 (10)	0.0586 (11)	0.0754 (12)	0.0143 (8)	0.0035 (8)	0.0188 (9)
C16	0.0472 (10)	0.0434 (9)	0.0674 (10)	0.0084 (7)	0.0058 (8)	0.0178 (8)
C17	0.0515 (9)	0.0404 (8)	0.0432 (8)	0.0041 (7)	0.0068 (7)	0.0026 (7)
C18	0.1029 (17)	0.0871 (15)	0.0472 (10)	−0.0074 (13)	−0.0152 (10)	0.0165 (10)
C19	0.0560 (10)	0.0487 (10)	0.0569 (10)	0.0157 (8)	0.0199 (8)	0.0045 (8)
C20	0.153 (3)	0.1032 (18)	0.0904 (17)	0.0530 (18)	0.0810 (18)	0.0205 (14)
C21	0.1208 (19)	0.0549 (11)	0.0772 (14)	0.0400 (12)	−0.0099 (13)	0.0101 (10)
C22	0.0435 (8)	0.0317 (7)	0.0391 (7)	0.0057 (6)	0.0063 (6)	0.0000 (6)
C23	0.0445 (9)	0.0430 (8)	0.0434 (8)	0.0083 (7)	0.0073 (6)	0.0042 (6)
C24	0.0519 (10)	0.0529 (10)	0.0541 (9)	0.0010 (8)	0.0181 (8)	0.0030 (8)
C25	0.0831 (13)	0.0392 (9)	0.0477 (9)	0.0033 (8)	0.0232 (9)	0.0035 (7)
C26	0.0863 (14)	0.0385 (8)	0.0514 (9)	0.0189 (9)	0.0104 (9)	0.0084 (7)
C27	0.0538 (10)	0.0401 (8)	0.0563 (9)	0.0140 (7)	0.0066 (7)	0.0046 (7)
C28	0.129 (2)	0.0605 (12)	0.0908 (16)	0.0013 (13)	0.0515 (15)	0.0203 (11)
O1	0.0382 (6)	0.0576 (7)	0.0956 (9)	0.0162 (5)	0.0070 (6)	0.0054 (6)
O2	0.1068 (11)	0.0500 (8)	0.0825 (9)	0.0275 (7)	0.0436 (8)	0.0008 (6)
O3	0.0830 (9)	0.0626 (8)	0.0690 (8)	0.0226 (7)	0.0432 (7)	0.0144 (6)
O4	0.0977 (10)	0.0488 (7)	0.0551 (7)	−0.0138 (7)	−0.0069 (7)	0.0012 (6)
O5	0.0773 (8)	0.0510 (7)	0.0415 (6)	−0.0017 (6)	−0.0036 (5)	0.0095 (5)
O6	0.0803 (10)	0.0613 (8)	0.0852 (9)	0.0335 (7)	−0.0090 (7)	0.0141 (7)

### (I) Dimethyl 4-(4-methoxyphenyl)-2-(4-methylphenyl)-1-oxo-1,2-dihydronaphthalene-2,3-dicarboxylate Geometric parameters (Å, º)

**Table:**

C1—C2	1.389 (2)	C17—O4	1.1990 (19)
C1—C6	1.397 (2)	C17—O5	1.3204 (19)
C1—C10	1.472 (2)	C18—O5	1.438 (2)
C2—C3	1.379 (2)	C18—H18A	0.9600
C2—H2	0.9300	C18—H18B	0.9600
C3—C4	1.369 (2)	C18—H18C	0.9600
C3—H3	0.9300	C19—O2	1.186 (2)
C4—C5	1.373 (2)	C19—O3	1.331 (2)
C4—H4	0.9300	C20—O3	1.444 (2)
C5—C6	1.383 (2)	C20—H20A	0.9600
C5—H5	0.9300	C20—H20B	0.9600
C6—C7	1.473 (2)	C20—H20C	0.9600
C7—O1	1.2134 (18)	C21—O6	1.412 (3)
C7—C8	1.531 (2)	C21—H21A	0.9600
C8—C9	1.522 (2)	C21—H21B	0.9600
C8—C19	1.535 (2)	C21—H21C	0.9600
C8—C22	1.542 (2)	C22—C23	1.380 (2)
C9—C10	1.342 (2)	C22—C27	1.383 (2)
C9—C17	1.487 (2)	C23—C24	1.384 (2)
C10—C11	1.487 (2)	C23—H23	0.9300
C11—C12	1.380 (2)	C24—C25	1.375 (3)
C11—C16	1.381 (2)	C24—H24	0.9300
C12—C13	1.384 (2)	C25—C26	1.379 (3)
C12—H12	0.9300	C25—C28	1.506 (3)
C13—C14	1.377 (3)	C26—C27	1.373 (2)
C13—H13	0.9300	C26—H26	0.9300
C14—O6	1.364 (2)	C27—H27	0.9300
C14—C15	1.374 (2)	C28—H28A	0.9600
C15—C16	1.374 (2)	C28—H28B	0.9600
C15—H15	0.9300	C28—H28C	0.9600
C16—H16	0.9300		
			
C2—C1—C6	117.75 (13)	O4—C17—O5	123.66 (15)
C2—C1—C10	122.02 (13)	O4—C17—C9	123.29 (14)
C6—C1—C10	120.21 (13)	O5—C17—C9	112.94 (13)
C3—C2—C1	121.18 (15)	O5—C18—H18A	109.5
C3—C2—H2	119.4	O5—C18—H18B	109.5
C1—C2—H2	119.4	H18A—C18—H18B	109.5
C4—C3—C2	120.32 (15)	O5—C18—H18C	109.5
C4—C3—H3	119.8	H18A—C18—H18C	109.5
C2—C3—H3	119.8	H18B—C18—H18C	109.5
C3—C4—C5	119.76 (15)	O2—C19—O3	124.44 (15)
C3—C4—H4	120.1	O2—C19—C8	126.23 (15)
C5—C4—H4	120.1	O3—C19—C8	109.33 (13)
C4—C5—C6	120.48 (16)	O3—C20—H20A	109.5
C4—C5—H5	119.8	O3—C20—H20B	109.5
C6—C5—H5	119.8	H20A—C20—H20B	109.5
C5—C6—C1	120.50 (14)	O3—C20—H20C	109.5
C5—C6—C7	119.92 (14)	H20A—C20—H20C	109.5
C1—C6—C7	119.58 (13)	H20B—C20—H20C	109.5
O1—C7—C6	122.60 (15)	O6—C21—H21A	109.5
O1—C7—C8	121.18 (14)	O6—C21—H21B	109.5
C6—C7—C8	116.07 (12)	H21A—C21—H21B	109.5
C9—C8—C7	110.59 (11)	O6—C21—H21C	109.5
C9—C8—C19	110.62 (12)	H21A—C21—H21C	109.5
C7—C8—C19	107.56 (12)	H21B—C21—H21C	109.5
C9—C8—C22	113.30 (12)	C23—C22—C27	118.00 (14)
C7—C8—C22	104.74 (11)	C23—C22—C8	122.47 (13)
C19—C8—C22	109.74 (12)	C27—C22—C8	119.53 (13)
C10—C9—C17	122.91 (13)	C22—C23—C24	120.40 (15)
C10—C9—C8	122.15 (13)	C22—C23—H23	119.8
C17—C9—C8	114.88 (12)	C24—C23—H23	119.8
C9—C10—C1	120.24 (13)	C25—C24—C23	121.70 (16)
C9—C10—C11	121.87 (13)	C25—C24—H24	119.2
C1—C10—C11	117.65 (12)	C23—C24—H24	119.2
C12—C11—C16	117.89 (14)	C24—C25—C26	117.36 (15)
C12—C11—C10	123.18 (14)	C24—C25—C28	121.8 (2)
C16—C11—C10	118.88 (13)	C26—C25—C28	120.81 (19)
C11—C12—C13	121.32 (15)	C27—C26—C25	121.52 (16)
C11—C12—H12	119.3	C27—C26—H26	119.2
C13—C12—H12	119.3	C25—C26—H26	119.2
C14—C13—C12	119.67 (15)	C26—C27—C22	120.94 (16)
C14—C13—H13	120.2	C26—C27—H27	119.5
C12—C13—H13	120.2	C22—C27—H27	119.5
O6—C14—C15	115.00 (16)	C25—C28—H28A	109.5
O6—C14—C13	125.43 (16)	C25—C28—H28B	109.5
C15—C14—C13	119.57 (15)	H28A—C28—H28B	109.5
C16—C15—C14	120.27 (17)	C25—C28—H28C	109.5
C16—C15—H15	119.9	H28A—C28—H28C	109.5
C14—C15—H15	119.9	H28B—C28—H28C	109.5
C15—C16—C11	121.27 (15)	C19—O3—C20	115.56 (15)
C15—C16—H16	119.4	C17—O5—C18	116.25 (13)
C11—C16—H16	119.4	C14—O6—C21	118.23 (17)
			
C6—C1—C2—C3	−0.6 (2)	C11—C12—C13—C14	1.2 (2)
C10—C1—C2—C3	−179.19 (14)	C12—C13—C14—O6	178.48 (15)
C1—C2—C3—C4	0.4 (3)	C12—C13—C14—C15	−0.7 (2)
C2—C3—C4—C5	0.3 (3)	O6—C14—C15—C16	−179.47 (16)
C3—C4—C5—C6	−0.7 (3)	C13—C14—C15—C16	−0.2 (3)
C4—C5—C6—C1	0.5 (2)	C14—C15—C16—C11	0.7 (3)
C4—C5—C6—C7	179.96 (15)	C12—C11—C16—C15	−0.2 (2)
C2—C1—C6—C5	0.1 (2)	C10—C11—C16—C15	177.47 (15)
C10—C1—C6—C5	178.74 (14)	C10—C9—C17—O4	−138.46 (18)
C2—C1—C6—C7	−179.29 (13)	C8—C9—C17—O4	38.7 (2)
C10—C1—C6—C7	−0.7 (2)	C10—C9—C17—O5	45.2 (2)
C5—C6—C7—O1	−20.2 (2)	C8—C9—C17—O5	−137.56 (14)
C1—C6—C7—O1	159.22 (15)	C9—C8—C19—O2	−135.20 (19)
C5—C6—C7—C8	155.47 (14)	C7—C8—C19—O2	103.9 (2)
C1—C6—C7—C8	−25.1 (2)	C22—C8—C19—O2	−9.5 (2)
O1—C7—C8—C9	−146.10 (15)	C9—C8—C19—O3	44.87 (18)
C6—C7—C8—C9	38.16 (17)	C7—C8—C19—O3	−76.01 (17)
O1—C7—C8—C19	−25.2 (2)	C22—C8—C19—O3	170.60 (13)
C6—C7—C8—C19	159.05 (13)	C9—C8—C22—C23	7.84 (19)
O1—C7—C8—C22	91.51 (17)	C7—C8—C22—C23	128.45 (14)
C6—C7—C8—C22	−84.24 (15)	C19—C8—C22—C23	−116.35 (15)
C7—C8—C9—C10	−29.46 (18)	C9—C8—C22—C27	−173.06 (12)
C19—C8—C9—C10	−148.52 (14)	C7—C8—C22—C27	−52.45 (16)
C22—C8—C9—C10	87.78 (16)	C19—C8—C22—C27	62.75 (17)
C7—C8—C9—C17	153.32 (12)	C27—C22—C23—C24	−2.6 (2)
C19—C8—C9—C17	34.26 (17)	C8—C22—C23—C24	176.56 (13)
C22—C8—C9—C17	−89.44 (14)	C22—C23—C24—C25	0.8 (2)
C17—C9—C10—C1	−177.56 (13)	C23—C24—C25—C26	1.7 (2)
C8—C9—C10—C1	5.4 (2)	C23—C24—C25—C28	−176.07 (17)
C17—C9—C10—C11	8.2 (2)	C24—C25—C26—C27	−2.5 (2)
C8—C9—C10—C11	−168.83 (13)	C28—C25—C26—C27	175.31 (17)
C2—C1—C10—C9	−170.09 (14)	C25—C26—C27—C22	0.8 (2)
C6—C1—C10—C9	11.4 (2)	C23—C22—C27—C26	1.8 (2)
C2—C1—C10—C11	4.4 (2)	C8—C22—C27—C26	−177.35 (14)
C6—C1—C10—C11	−174.13 (12)	O2—C19—O3—C20	2.7 (3)
C9—C10—C11—C12	−117.13 (17)	C8—C19—O3—C20	−177.38 (18)
C1—C10—C11—C12	68.45 (18)	O4—C17—O5—C18	1.3 (3)
C9—C10—C11—C16	65.36 (19)	C9—C17—O5—C18	177.56 (16)
C1—C10—C11—C16	−109.06 (16)	C15—C14—O6—C21	−166.54 (17)
C16—C11—C12—C13	−0.7 (2)	C13—C14—O6—C21	14.3 (2)
C10—C11—C12—C13	−178.28 (14)		

### (I) Dimethyl 4-(4-methoxyphenyl)-2-(4-methylphenyl)-1-oxo-1,2-dihydronaphthalene-2,3-dicarboxylate Hydrogen-bond geometry (Å, º)

*Cg*4 is the centroid of the C22–C27 benzene ring .

**Table:**

*D*—H···*A*	*D*—H	H···*A*	*D*···*A*	*D*—H···*A*
C27—H27···O1	0.93	2.52	3.109 (2)	121
C16—H16···O1^i^	0.93	2.52	3.344 (3)	148
C3—H3···*Cg*4^ii^	0.93	2.78	3.656 (2)	157

Symmetry codes: (i) *x*−1, *y*, *z*; (ii) −*x*+2, −*y*+1, −*z*+1.

### (II) Dimethyl 1-oxo-2-(pyren-4-yl)-4-(thiophen-2-yl)-1,2-dihydronaphthalene-2,3-dicarboxylate Crystal data

**Table:**

C_34_H_22_O_5_S	*F*(000) = 1128
*M**_r_* = 542.58	*D*_x_ = 1.420 Mg m^−^^3^
Monoclinic, *P*2_1_/*n*	Mo *K*α radiation, λ = 0.71073 Å
Hall symbol: -P 2yn	Cell parameters from 4457 reflections
*a* = 10.9268 (10) Å	θ = 2.0–25.0°
*b* = 18.9670 (14) Å	µ = 0.17 mm^−^^1^
*c* = 12.2628 (9) Å	*T* = 296 K
β = 93.030 (2)°	Block, colourless
*V* = 2537.9 (4) Å^3^	0.25 × 0.25 × 0.20 mm
*Z* = 4	

### (II) Dimethyl 1-oxo-2-(pyren-4-yl)-4-(thiophen-2-yl)-1,2-dihydronaphthalene-2,3-dicarboxylate Data collection

**Table:**

Bruker Kappa APEXII CCD diffractometer	4457 independent reflections
Radiation source: fine-focus sealed tube	3341 reflections with *I* > 2σ(*I*)
Graphite monochromator	*R*_int_ = 0.031
ω & φ scans	θ_max_ = 25.0°, θ_min_ = 2.0°
Absorption correction: multi-scan (SADABS; Bruker, 2008)	*h* = −12→12
*T*_min_ = 0.958, *T*_max_ = 0.966	*k* = −22→22
21576 measured reflections	*l* = −14→14

### (II) Dimethyl 1-oxo-2-(pyren-4-yl)-4-(thiophen-2-yl)-1,2-dihydronaphthalene-2,3-dicarboxylate Refinement

**Table:**

Refinement on *F*^2^	Primary atom site location: structure-invariant direct methods
Least-squares matrix: full	Secondary atom site location: difference Fourier map
*R*[*F*^2^ > 2σ(*F*^2^)] = 0.039	Hydrogen site location: inferred from neighbouring sites
*wR*(*F*^2^) = 0.105	H-atom parameters constrained
*S* = 1.04	*w* = 1/[σ^2^(*F*_o_^2^) + (0.042*P*)^2^ + 1.1182*P*] where *P* = (*F*_o_^2^ + 2*F*_c_^2^)/3
4457 reflections	(Δ/σ)_max_ = 0.003
400 parameters	Δρ_max_ = 0.22 e Å^−^^3^
56 restraints	Δρ_min_ = −0.22 e Å^−^^3^

### (II) Dimethyl 1-oxo-2-(pyren-4-yl)-4-(thiophen-2-yl)-1,2-dihydronaphthalene-2,3-dicarboxylate Special details

**Table:**

Geometry. All esds (except the esd in the dihedral angle between two l.s. planes) are estimated using the full covariance matrix. The cell esds are taken into account individually in the estimation of esds in distances, angles and torsion angles; correlations between esds in cell parameters are only used when they are defined by crystal symmetry. An approximate (isotropic) treatment of cell esds is used for estimating esds involving l.s. planes.
Refinement. Refinement of F^2^ against ALL reflections. The weighted R-factor wR and goodness of fit S are based on F^2^, conventional R-factors R are based on F, with F set to zero for negative F^2^. The threshold expression of F^2^ > 2sigma(F^2^) is used only for calculating R-factors(gt) etc. and is not relevant to the choice of reflections for refinement. R-factors based on F^2^ are statistically about twice as large as those based on F, and R- factors based on ALL data will be even larger.

### (II) Dimethyl 1-oxo-2-(pyren-4-yl)-4-(thiophen-2-yl)-1,2-dihydronaphthalene-2,3-dicarboxylate Fractional atomic coordinates and isotropic or equivalent isotropic displacement parameters (Å^2^)

**Table:**

	*x*	*y*	*z*	*U*_iso_*/*U*_eq_	Occ. (<1)
C1	0.53176 (18)	0.11069 (10)	0.09837 (15)	0.0320 (5)	
C2	0.4292 (2)	0.11714 (12)	0.02630 (16)	0.0416 (5)	
H2	0.3605	0.0895	0.0364	0.050*	
C3	0.4284 (2)	0.16390 (12)	−0.05966 (17)	0.0478 (6)	
H3	0.3601	0.1664	−0.1080	0.057*	
C4	0.5269 (2)	0.20675 (12)	−0.07487 (18)	0.0498 (6)	
H4	0.5261	0.2375	−0.1340	0.060*	
C5	0.6268 (2)	0.20404 (11)	−0.00227 (17)	0.0452 (6)	
H5	0.6923	0.2346	−0.0106	0.054*	
C6	0.63113 (19)	0.15603 (10)	0.08371 (15)	0.0351 (5)	
C7	0.73736 (19)	0.15549 (11)	0.16206 (16)	0.0376 (5)	
C8	0.76155 (18)	0.08696 (10)	0.22786 (15)	0.0321 (5)	
C9	0.64414 (18)	0.04512 (9)	0.24500 (15)	0.0297 (4)	
C10	0.53834 (18)	0.05803 (10)	0.18734 (15)	0.0299 (4)	
C11	0.42509 (18)	0.02128 (10)	0.21535 (16)	0.0336 (5)	
C15	0.84101 (19)	0.04475 (11)	0.15007 (16)	0.0392 (5)	
C16	0.8909 (2)	−0.06625 (13)	0.0818 (2)	0.0595 (7)	
H16A	0.8763	−0.0497	0.0083	0.089*	
H16B	0.8652	−0.1145	0.0865	0.089*	
H16C	0.9768	−0.0629	0.1021	0.089*	
C17	0.65383 (19)	−0.01355 (10)	0.32741 (17)	0.0355 (5)	
C18	0.5913 (3)	−0.12957 (12)	0.3641 (2)	0.0642 (7)	
H18A	0.6683	−0.1403	0.4018	0.096*	
H18B	0.5611	−0.1707	0.3258	0.096*	
H18C	0.5333	−0.1152	0.4158	0.096*	
C19	0.83524 (18)	0.10328 (10)	0.33498 (15)	0.0321 (4)	
C20	0.94999 (19)	0.07443 (11)	0.35762 (18)	0.0400 (5)	
H20	0.9840	0.0456	0.3058	0.048*	
C21	1.01527 (19)	0.08713 (11)	0.45472 (18)	0.0417 (5)	
H21	1.0915	0.0661	0.4677	0.050*	
C22	0.96935 (18)	0.13071 (10)	0.53330 (16)	0.0351 (5)	
C23	1.0356 (2)	0.14591 (12)	0.63475 (18)	0.0458 (6)	
H23	1.1102	0.1236	0.6508	0.055*	
C24	0.9928 (2)	0.19113 (12)	0.70620 (18)	0.0472 (6)	
H24	1.0394	0.2005	0.7702	0.057*	
C25	0.8774 (2)	0.22576 (11)	0.68799 (16)	0.0383 (5)	
C26	0.8312 (2)	0.27434 (12)	0.76016 (18)	0.0494 (6)	
H26	0.8772	0.2855	0.8238	0.059*	
C27	0.7194 (2)	0.30626 (13)	0.73977 (19)	0.0507 (6)	
H27	0.6915	0.3390	0.7892	0.061*	
C28	0.6483 (2)	0.29028 (11)	0.64711 (17)	0.0425 (5)	
H28	0.5721	0.3115	0.6349	0.051*	
C29	0.69065 (18)	0.24205 (10)	0.57106 (15)	0.0333 (5)	
C30	0.62192 (18)	0.22445 (10)	0.47291 (16)	0.0366 (5)	
H30	0.5443	0.2438	0.4602	0.044*	
C31	0.66595 (18)	0.18065 (10)	0.39807 (16)	0.0346 (5)	
H31	0.6182	0.1712	0.3347	0.042*	
C32	0.78410 (17)	0.14808 (10)	0.41282 (15)	0.0301 (4)	
C33	0.85277 (17)	0.16242 (10)	0.51215 (15)	0.0305 (4)	
C34	0.80620 (18)	0.20967 (10)	0.59065 (15)	0.0315 (4)	
O1	0.80805 (16)	0.20458 (8)	0.17246 (13)	0.0578 (5)	
O2	0.91056 (18)	0.07160 (9)	0.09104 (16)	0.0783 (6)	
O3	0.82234 (14)	−0.02357 (8)	0.15510 (12)	0.0491 (4)	
O4	0.69985 (18)	−0.00824 (9)	0.41768 (13)	0.0651 (5)	
O5	0.60807 (14)	−0.07340 (7)	0.28718 (12)	0.0445 (4)	
S1	0.37700 (13)	0.02866 (9)	0.34494 (12)	0.0438 (3)	0.69
C12	0.3568 (12)	−0.0254 (7)	0.1509 (9)	0.064 (3)	0.69
H12	0.3719	−0.0367	0.0790	0.076*	0.69
C13	0.2563 (11)	−0.0549 (7)	0.2127 (7)	0.0570 (16)	0.69
H13	0.1993	−0.0878	0.1856	0.068*	0.69
C14	0.2593 (8)	−0.0272 (5)	0.3137 (6)	0.0508 (18)	0.69
H14	0.2005	−0.0381	0.3633	0.061*	0.69
S1'	0.3399 (8)	−0.0324 (4)	0.1337 (6)	0.0532 (11)	0.31
C12'	0.3651 (16)	0.0294 (11)	0.3107 (10)	0.077 (5)	0.31
H12'	0.3839	0.0669	0.3574	0.092*	0.31
C13'	0.270 (2)	−0.0228 (15)	0.3377 (19)	0.064 (4)	0.31
H13'	0.2373	−0.0337	0.4040	0.077*	0.31
C14'	0.245 (3)	−0.0501 (17)	0.2351 (16)	0.055 (3)	0.31
H14'	0.1768	−0.0784	0.2214	0.066*	0.31

### (II) Dimethyl 1-oxo-2-(pyren-4-yl)-4-(thiophen-2-yl)-1,2-dihydronaphthalene-2,3-dicarboxylate Atomic displacement parameters (Å^2^)

**Table:**

	*U*^11^	*U*^22^	*U*^33^	*U*^12^	*U*^13^	*U*^23^
C1	0.0379 (12)	0.0319 (10)	0.0268 (10)	−0.0001 (9)	0.0069 (9)	−0.0045 (8)
C2	0.0402 (13)	0.0480 (13)	0.0368 (12)	−0.0005 (10)	0.0029 (10)	0.0000 (10)
C3	0.0518 (15)	0.0551 (14)	0.0360 (12)	0.0084 (12)	−0.0025 (10)	0.0018 (11)
C4	0.0671 (17)	0.0462 (13)	0.0363 (12)	0.0051 (12)	0.0041 (12)	0.0081 (10)
C5	0.0577 (15)	0.0386 (12)	0.0398 (12)	−0.0069 (11)	0.0081 (11)	0.0050 (10)
C6	0.0437 (12)	0.0311 (11)	0.0310 (11)	−0.0019 (9)	0.0056 (9)	−0.0008 (9)
C7	0.0419 (12)	0.0364 (11)	0.0352 (11)	−0.0094 (10)	0.0084 (9)	−0.0010 (9)
C8	0.0318 (11)	0.0348 (11)	0.0304 (10)	−0.0047 (9)	0.0060 (8)	−0.0018 (8)
C9	0.0353 (11)	0.0288 (10)	0.0257 (10)	−0.0042 (8)	0.0069 (8)	−0.0049 (8)
C10	0.0337 (11)	0.0302 (10)	0.0261 (10)	−0.0023 (9)	0.0062 (8)	−0.0045 (8)
C11	0.0324 (11)	0.0337 (11)	0.0351 (11)	−0.0021 (9)	0.0053 (9)	−0.0010 (9)
C15	0.0368 (12)	0.0459 (13)	0.0355 (11)	−0.0059 (10)	0.0080 (10)	−0.0035 (10)
C16	0.0592 (16)	0.0565 (15)	0.0646 (16)	0.0104 (13)	0.0199 (13)	−0.0144 (13)
C17	0.0347 (11)	0.0372 (12)	0.0349 (12)	−0.0044 (9)	0.0057 (9)	0.0014 (9)
C18	0.0783 (19)	0.0404 (13)	0.0748 (18)	−0.0105 (13)	0.0119 (15)	0.0184 (13)
C19	0.0314 (11)	0.0302 (10)	0.0346 (11)	−0.0046 (9)	0.0025 (9)	−0.0009 (8)
C20	0.0345 (12)	0.0371 (11)	0.0488 (13)	0.0013 (9)	0.0049 (10)	−0.0054 (10)
C21	0.0278 (11)	0.0399 (12)	0.0567 (14)	0.0032 (9)	−0.0037 (10)	0.0009 (10)
C22	0.0299 (11)	0.0310 (11)	0.0437 (12)	−0.0026 (9)	−0.0037 (9)	0.0029 (9)
C23	0.0347 (12)	0.0481 (13)	0.0529 (14)	−0.0006 (10)	−0.0138 (11)	0.0051 (11)
C24	0.0488 (14)	0.0503 (14)	0.0406 (12)	−0.0083 (11)	−0.0159 (11)	0.0001 (11)
C25	0.0412 (12)	0.0385 (11)	0.0345 (11)	−0.0091 (10)	−0.0050 (9)	0.0015 (9)
C26	0.0578 (16)	0.0552 (14)	0.0346 (12)	−0.0111 (12)	−0.0027 (11)	−0.0090 (11)
C27	0.0560 (16)	0.0532 (14)	0.0437 (13)	−0.0039 (12)	0.0099 (12)	−0.0147 (11)
C28	0.0395 (12)	0.0454 (12)	0.0432 (12)	0.0003 (10)	0.0065 (10)	−0.0029 (10)
C29	0.0345 (12)	0.0323 (11)	0.0332 (11)	−0.0048 (9)	0.0031 (9)	−0.0003 (9)
C30	0.0284 (11)	0.0394 (12)	0.0418 (12)	0.0037 (9)	−0.0007 (9)	0.0019 (9)
C31	0.0330 (11)	0.0367 (11)	0.0334 (11)	−0.0001 (9)	−0.0052 (9)	−0.0013 (9)
C32	0.0297 (10)	0.0270 (10)	0.0334 (10)	−0.0035 (8)	0.0002 (8)	0.0011 (8)
C33	0.0298 (11)	0.0273 (10)	0.0340 (11)	−0.0057 (8)	−0.0027 (8)	0.0036 (8)
C34	0.0335 (11)	0.0290 (10)	0.0317 (10)	−0.0065 (9)	0.0002 (9)	0.0033 (8)
O1	0.0631 (11)	0.0459 (9)	0.0633 (11)	−0.0261 (9)	−0.0084 (9)	0.0094 (8)
O2	0.0911 (15)	0.0622 (11)	0.0875 (13)	−0.0153 (11)	0.0616 (12)	−0.0073 (10)
O3	0.0542 (10)	0.0388 (8)	0.0567 (10)	−0.0007 (7)	0.0249 (8)	−0.0075 (7)
O4	0.0869 (14)	0.0657 (11)	0.0405 (10)	−0.0292 (10)	−0.0160 (9)	0.0160 (8)
O5	0.0590 (10)	0.0298 (8)	0.0451 (8)	−0.0050 (7)	0.0070 (7)	0.0016 (6)
S1	0.0458 (6)	0.0438 (6)	0.0437 (7)	0.0005 (5)	0.0210 (6)	0.0062 (5)
C12	0.041 (5)	0.068 (6)	0.083 (6)	−0.010 (3)	0.011 (4)	0.009 (4)
C13	0.037 (3)	0.052 (3)	0.083 (4)	−0.013 (2)	0.006 (3)	0.001 (3)
C14	0.038 (3)	0.055 (3)	0.062 (5)	−0.004 (2)	0.026 (3)	0.014 (3)
S1'	0.044 (2)	0.0488 (18)	0.066 (2)	−0.0163 (16)	−0.0030 (15)	−0.0054 (15)
C12'	0.100 (8)	0.062 (7)	0.068 (9)	−0.009 (6)	−0.009 (7)	0.003 (7)
C13'	0.070 (7)	0.066 (7)	0.057 (6)	0.005 (5)	−0.001 (5)	0.001 (6)
C14'	0.048 (7)	0.057 (6)	0.058 (6)	−0.012 (5)	0.004 (5)	0.001 (5)

### (II) Dimethyl 1-oxo-2-(pyren-4-yl)-4-(thiophen-2-yl)-1,2-dihydronaphthalene-2,3-dicarboxylate Geometric parameters (Å, º)

**Table:**

C1—C2	1.395 (3)	C20—C21	1.376 (3)
C1—C6	1.404 (3)	C20—H20	0.9300
C1—C10	1.478 (3)	C21—C22	1.384 (3)
C2—C3	1.377 (3)	C21—H21	0.9300
C2—H2	0.9300	C22—C33	1.420 (3)
C3—C4	1.369 (3)	C22—C23	1.435 (3)
C3—H3	0.9300	C23—C24	1.329 (3)
C4—C5	1.373 (3)	C23—H23	0.9300
C4—H4	0.9300	C24—C25	1.429 (3)
C5—C6	1.392 (3)	C24—H24	0.9300
C5—H5	0.9300	C25—C26	1.391 (3)
C6—C7	1.467 (3)	C25—C34	1.423 (3)
C7—O1	1.212 (2)	C26—C27	1.375 (3)
C7—C8	1.545 (3)	C26—H26	0.9300
C8—C9	1.533 (3)	C27—C28	1.376 (3)
C8—C19	1.536 (3)	C27—H27	0.9300
C8—C15	1.547 (3)	C28—C29	1.402 (3)
C9—C10	1.345 (3)	C28—H28	0.9300
C9—C17	1.503 (3)	C29—C34	1.413 (3)
C10—C11	1.477 (3)	C29—C30	1.424 (3)
C11—C12'	1.379 (9)	C30—C31	1.346 (3)
C11—C12	1.380 (7)	C30—H30	0.9300
C11—S1'	1.676 (5)	C31—C32	1.434 (3)
C11—S1	1.706 (2)	C31—H31	0.9300
C15—O2	1.191 (2)	C32—C33	1.423 (3)
C15—O3	1.314 (2)	C33—C34	1.429 (3)
C16—O3	1.448 (2)	S1—C14	1.695 (6)
C16—H16A	0.9600	C12—C13	1.477 (8)
C16—H16B	0.9600	C12—H12	0.9300
C16—H16C	0.9600	C13—C14	1.345 (6)
C17—O4	1.196 (2)	C13—H13	0.9300
C17—O5	1.325 (2)	C14—H14	0.9300
C18—O5	1.441 (3)	S1'—C14'	1.691 (9)
C18—H18A	0.9600	C12'—C13'	1.483 (10)
C18—H18B	0.9600	C12'—H12'	0.9300
C18—H18C	0.9600	C13'—C14'	1.375 (10)
C19—C20	1.383 (3)	C13'—H13'	0.9300
C19—C32	1.415 (3)	C14'—H14'	0.9300
			
C2—C1—C6	117.75 (18)	C20—C21—C22	121.10 (19)
C2—C1—C10	122.20 (18)	C20—C21—H21	119.4
C6—C1—C10	120.04 (18)	C22—C21—H21	119.4
C3—C2—C1	120.9 (2)	C21—C22—C33	118.72 (18)
C3—C2—H2	119.5	C21—C22—C23	122.59 (19)
C1—C2—H2	119.5	C33—C22—C23	118.68 (19)
C4—C3—C2	120.9 (2)	C24—C23—C22	121.4 (2)
C4—C3—H3	119.6	C24—C23—H23	119.3
C2—C3—H3	119.6	C22—C23—H23	119.3
C3—C4—C5	119.6 (2)	C23—C24—C25	122.2 (2)
C3—C4—H4	120.2	C23—C24—H24	118.9
C5—C4—H4	120.2	C25—C24—H24	118.9
C4—C5—C6	120.6 (2)	C26—C25—C34	118.4 (2)
C4—C5—H5	119.7	C26—C25—C24	123.5 (2)
C6—C5—H5	119.7	C34—C25—C24	118.11 (19)
C5—C6—C1	120.2 (2)	C27—C26—C25	121.7 (2)
C5—C6—C7	119.62 (19)	C27—C26—H26	119.2
C1—C6—C7	120.15 (17)	C25—C26—H26	119.2
O1—C7—C6	122.77 (19)	C26—C27—C28	120.7 (2)
O1—C7—C8	120.05 (19)	C26—C27—H27	119.6
C6—C7—C8	117.08 (17)	C28—C27—H27	119.6
C9—C8—C19	113.08 (15)	C27—C28—C29	120.1 (2)
C9—C8—C7	112.70 (16)	C27—C28—H28	120.0
C19—C8—C7	110.16 (15)	C29—C28—H28	120.0
C9—C8—C15	108.53 (15)	C28—C29—C34	119.58 (18)
C19—C8—C15	110.24 (16)	C28—C29—C30	122.49 (19)
C7—C8—C15	101.51 (15)	C34—C29—C30	117.93 (17)
C10—C9—C17	121.08 (17)	C31—C30—C29	121.97 (19)
C10—C9—C8	122.41 (17)	C31—C30—H30	119.0
C17—C9—C8	116.48 (17)	C29—C30—H30	119.0
C9—C10—C11	119.94 (17)	C30—C31—C32	122.13 (19)
C9—C10—C1	121.19 (17)	C30—C31—H31	118.9
C11—C10—C1	118.83 (17)	C32—C31—H31	118.9
C12'—C11—C12	106.9 (10)	C19—C32—C33	118.90 (17)
C12'—C11—C10	125.9 (8)	C19—C32—C31	123.96 (18)
C12—C11—C10	127.2 (5)	C33—C32—C31	117.14 (17)
C12'—C11—S1'	107.5 (8)	C22—C33—C32	120.18 (18)
C10—C11—S1'	126.4 (3)	C22—C33—C34	119.39 (17)
C12—C11—S1	113.7 (5)	C32—C33—C34	120.43 (17)
C10—C11—S1	118.77 (15)	C29—C34—C25	119.55 (18)
S1'—C11—S1	114.7 (3)	C29—C34—C33	120.33 (17)
O2—C15—O3	123.8 (2)	C25—C34—C33	120.10 (18)
O2—C15—C8	123.4 (2)	C15—O3—C16	115.83 (17)
O3—C15—C8	112.83 (16)	C17—O5—C18	116.64 (17)
O3—C16—H16A	109.5	C14—S1—C11	90.3 (3)
O3—C16—H16B	109.5	C11—C12—C13	110.1 (10)
H16A—C16—H16B	109.5	C11—C12—H12	124.9
O3—C16—H16C	109.5	C13—C12—H12	124.9
H16A—C16—H16C	109.5	C14—C13—C12	110.0 (9)
H16B—C16—H16C	109.5	C14—C13—H13	125.0
O4—C17—O5	123.28 (19)	C12—C13—H13	125.0
O4—C17—C9	124.84 (19)	C13—C14—S1	115.7 (7)
O5—C17—C9	111.85 (17)	C13—C14—H14	122.2
O5—C18—H18A	109.5	S1—C14—H14	122.2
O5—C18—H18B	109.5	C11—S1'—C14'	91.3 (11)
H18A—C18—H18B	109.5	C11—C12'—C13'	119.0 (16)
O5—C18—H18C	109.5	C11—C12'—H12'	120.5
H18A—C18—H18C	109.5	C13'—C12'—H12'	120.5
H18B—C18—H18C	109.5	C14'—C13'—C12'	99 (2)
C20—C19—C32	119.24 (18)	C14'—C13'—H13'	130.6
C20—C19—C8	121.39 (17)	C12'—C13'—H13'	130.6
C32—C19—C8	119.36 (17)	C13'—C14'—S1'	120 (2)
C21—C20—C19	121.83 (19)	C13'—C14'—H14'	120.0
C21—C20—H20	119.1	S1'—C14'—H14'	120.0
C19—C20—H20	119.1		
			
C6—C1—C2—C3	3.3 (3)	C33—C22—C23—C24	−3.1 (3)
C10—C1—C2—C3	−176.23 (19)	C22—C23—C24—C25	1.7 (3)
C1—C2—C3—C4	−1.9 (3)	C23—C24—C25—C26	−178.7 (2)
C2—C3—C4—C5	−1.3 (3)	C23—C24—C25—C34	1.2 (3)
C3—C4—C5—C6	2.8 (3)	C34—C25—C26—C27	0.4 (3)
C4—C5—C6—C1	−1.3 (3)	C24—C25—C26—C27	−179.7 (2)
C4—C5—C6—C7	−178.3 (2)	C25—C26—C27—C28	0.8 (4)
C2—C1—C6—C5	−1.8 (3)	C26—C27—C28—C29	−1.3 (3)
C10—C1—C6—C5	177.82 (18)	C27—C28—C29—C34	0.5 (3)
C2—C1—C6—C7	175.18 (18)	C27—C28—C29—C30	−178.9 (2)
C10—C1—C6—C7	−5.2 (3)	C28—C29—C30—C31	176.80 (19)
C5—C6—C7—O1	18.2 (3)	C34—C29—C30—C31	−2.6 (3)
C1—C6—C7—O1	−158.8 (2)	C29—C30—C31—C32	0.9 (3)
C5—C6—C7—C8	−158.20 (18)	C20—C19—C32—C33	0.3 (3)
C1—C6—C7—C8	24.8 (3)	C8—C19—C32—C33	179.53 (16)
O1—C7—C8—C9	154.96 (19)	C20—C19—C32—C31	−179.83 (18)
C6—C7—C8—C9	−28.6 (2)	C8—C19—C32—C31	−0.6 (3)
O1—C7—C8—C19	27.7 (3)	C30—C31—C32—C19	−178.20 (19)
C6—C7—C8—C19	−155.88 (16)	C30—C31—C32—C33	1.7 (3)
O1—C7—C8—C15	−89.1 (2)	C21—C22—C33—C32	1.4 (3)
C6—C7—C8—C15	87.3 (2)	C23—C22—C33—C32	−179.49 (18)
C19—C8—C9—C10	140.57 (18)	C21—C22—C33—C34	−177.61 (18)
C7—C8—C9—C10	14.8 (2)	C23—C22—C33—C34	1.5 (3)
C15—C8—C9—C10	−96.8 (2)	C19—C32—C33—C22	−1.5 (3)
C19—C8—C9—C17	−41.6 (2)	C31—C32—C33—C22	178.61 (17)
C7—C8—C9—C17	−167.35 (15)	C19—C32—C33—C34	177.44 (17)
C15—C8—C9—C17	81.0 (2)	C31—C32—C33—C34	−2.4 (3)
C17—C9—C10—C11	8.8 (3)	C28—C29—C34—C25	0.8 (3)
C8—C9—C10—C11	−173.51 (16)	C30—C29—C34—C25	−179.80 (17)
C17—C9—C10—C1	−173.60 (16)	C28—C29—C34—C33	−177.67 (17)
C8—C9—C10—C1	4.1 (3)	C30—C29—C34—C33	1.7 (3)
C2—C1—C10—C9	169.55 (18)	C26—C25—C34—C29	−1.2 (3)
C6—C1—C10—C9	−10.0 (3)	C24—C25—C34—C29	178.88 (18)
C2—C1—C10—C11	−12.8 (3)	C26—C25—C34—C33	177.23 (18)
C6—C1—C10—C11	167.65 (17)	C24—C25—C34—C33	−2.6 (3)
C9—C10—C11—C12'	66.1 (11)	C22—C33—C34—C29	179.72 (17)
C1—C10—C11—C12'	−111.6 (10)	C32—C33—C34—C29	0.8 (3)
C9—C10—C11—C12	−116.3 (9)	C22—C33—C34—C25	1.3 (3)
C1—C10—C11—C12	66.0 (9)	C32—C33—C34—C25	−177.69 (17)
C9—C10—C11—S1'	−119.4 (5)	O2—C15—O3—C16	−1.0 (3)
C1—C10—C11—S1'	62.9 (5)	C8—C15—O3—C16	178.13 (18)
C9—C10—C11—S1	56.8 (2)	O4—C17—O5—C18	13.1 (3)
C1—C10—C11—S1	−120.94 (18)	C9—C17—O5—C18	−168.72 (18)
C9—C8—C15—O2	151.5 (2)	C12'—C11—S1—C14	48 (5)
C19—C8—C15—O2	−84.2 (3)	C12—C11—S1—C14	−3.0 (9)
C7—C8—C15—O2	32.5 (3)	C10—C11—S1—C14	−177.0 (4)
C9—C8—C15—O3	−27.7 (2)	S1'—C11—S1—C14	−0.4 (6)
C19—C8—C15—O3	96.7 (2)	C12'—C11—C12—C13	−6.8 (16)
C7—C8—C15—O3	−146.61 (18)	C10—C11—C12—C13	175.2 (8)
C10—C9—C17—O4	−133.5 (2)	S1'—C11—C12—C13	−111 (20)
C8—C9—C17—O4	48.6 (3)	S1—C11—C12—C13	1.8 (14)
C10—C9—C17—O5	48.3 (2)	C11—C12—C13—C14	0.7 (17)
C8—C9—C17—O5	−129.53 (18)	C12—C13—C14—S1	−3.0 (16)
C9—C8—C19—C20	114.1 (2)	C11—S1—C14—C13	3.5 (10)
C7—C8—C19—C20	−118.8 (2)	C12'—C11—S1'—C14'	−8.1 (16)
C15—C8—C19—C20	−7.6 (2)	C12—C11—S1'—C14'	68 (19)
C9—C8—C19—C32	−65.1 (2)	C10—C11—S1'—C14'	176.6 (12)
C7—C8—C19—C32	62.0 (2)	S1—C11—S1'—C14'	0.3 (14)
C15—C8—C19—C32	173.17 (17)	C12—C11—C12'—C13'	16 (2)
C32—C19—C20—C21	1.1 (3)	C10—C11—C12'—C13'	−165.9 (17)
C8—C19—C20—C21	−178.12 (18)	S1'—C11—C12'—C13'	19 (2)
C19—C20—C21—C22	−1.3 (3)	S1—C11—C12'—C13'	−115 (6)
C20—C21—C22—C33	0.0 (3)	C11—C12'—C13'—C14'	−20 (3)
C20—C21—C22—C23	−179.1 (2)	C12'—C13'—C14'—S1'	13 (3)
C21—C22—C23—C24	176.0 (2)	C11—S1'—C14'—C13'	−4 (3)

### (III) Ethyl 4-oxo-3-phenyl-1,3-bis(thiophen-2-yl)-3,4-dihydronaphthalene-2-carboxylate Crystal data

**Table:**

C_27_H_20_O_3_S_2_	*F*(000) = 952
*M**_r_* = 456.55	*D*_x_ = 1.340 Mg m^−^^3^
Monoclinic, *P*2_1_/*c*	Mo *K*α radiation, λ = 0.71073 Å
Hall symbol: -P 2ybc	Cell parameters from 4110 reflections
*a* = 12.1263 (11) Å	θ = 2.4–25.4°
*b* = 11.8009 (11) Å	µ = 0.26 mm^−^^1^
*c* = 16.0657 (13) Å	*T* = 296 K
β = 100.181 (2)°	Block, green
*V* = 2262.8 (3) Å^3^	0.25 × 0.25 × 0.15 mm
*Z* = 4	

### (III) Ethyl 4-oxo-3-phenyl-1,3-bis(thiophen-2-yl)-3,4-dihydronaphthalene-2-carboxylate Data collection

**Table:**

Bruker Kappa APEXII CCD diffractometer	4110 independent reflections
Radiation source: fine-focus sealed tube	2685 reflections with *I* > 2σ(*I*)
Graphite monochromator	*R*_int_ = 0.044
ω & φ scans	θ_max_ = 25.4°, θ_min_ = 2.4°
Absorption correction: multi-scan (SADABS; Bruker, 2008)	*h* = −14→14
*T*_min_ = 0.937, *T*_max_ = 0.962	*k* = −14→14
29901 measured reflections	*l* = −19→18

### (III) Ethyl 4-oxo-3-phenyl-1,3-bis(thiophen-2-yl)-3,4-dihydronaphthalene-2-carboxylate Refinement

**Table:**

Refinement on *F*^2^	Primary atom site location: structure-invariant direct methods
Least-squares matrix: full	Secondary atom site location: difference Fourier map
*R*[*F*^2^ > 2σ(*F*^2^)] = 0.061	Hydrogen site location: inferred from neighbouring sites
*wR*(*F*^2^) = 0.147	H-atom parameters constrained
*S* = 1.09	*w* = 1/[σ^2^(*F*_o_^2^) + (0.0338*P*)^2^ + 2.9594*P*] where *P* = (*F*_o_^2^ + 2*F*_c_^2^)/3
4110 reflections	(Δ/σ)_max_ = 0.004
364 parameters	Δρ_max_ = 0.24 e Å^−^^3^
100 restraints	Δρ_min_ = −0.24 e Å^−^^3^

### (III) Ethyl 4-oxo-3-phenyl-1,3-bis(thiophen-2-yl)-3,4-dihydronaphthalene-2-carboxylate Special details

**Table:**

Geometry. All esds (except the esd in the dihedral angle between two l.s. planes) are estimated using the full covariance matrix. The cell esds are taken into account individually in the estimation of esds in distances, angles and torsion angles; correlations between esds in cell parameters are only used when they are defined by crystal symmetry. An approximate (isotropic) treatment of cell esds is used for estimating esds involving l.s. planes.
Refinement. Refinement of F^2^ against ALL reflections. The weighted R-factor wR and goodness of fit S are based on F^2^, conventional R-factors R are based on F, with F set to zero for negative F^2^. The threshold expression of F^2^ > 2sigma(F^2^) is used only for calculating R-factors(gt) etc. and is not relevant to the choice of reflections for refinement. R-factors based on F^2^ are statistically about twice as large as those based on F, and R- factors based on ALL data will be even larger.

### (III) Ethyl 4-oxo-3-phenyl-1,3-bis(thiophen-2-yl)-3,4-dihydronaphthalene-2-carboxylate Fractional atomic coordinates and isotropic or equivalent isotropic displacement parameters (Å^2^)

**Table:**

	*x*	*y*	*z*	*U*_iso_*/*U*_eq_	Occ. (<1)
C7	0.6147 (3)	0.7554 (3)	0.11948 (19)	0.0431 (7)	
C6	0.5745 (2)	0.7440 (3)	0.02783 (18)	0.0395 (7)	
C5	0.4953 (3)	0.6615 (3)	−0.0013 (2)	0.0520 (8)	
H5	0.4686	0.6143	0.0371	0.062*	
C4	0.4560 (3)	0.6491 (3)	−0.0866 (2)	0.0601 (10)	
H4	0.4034	0.5934	−0.1059	0.072*	
C3	0.4950 (3)	0.7196 (3)	−0.1430 (2)	0.0576 (9)	
H3	0.4687	0.7112	−0.2006	0.069*	
C2	0.5725 (3)	0.8026 (3)	−0.11538 (19)	0.0481 (8)	
H2	0.5976	0.8499	−0.1544	0.058*	
C1	0.6138 (2)	0.8164 (3)	−0.02929 (18)	0.0370 (7)	
C10	0.6948 (2)	0.9062 (2)	0.00282 (17)	0.0353 (7)	
C9	0.7444 (2)	0.9089 (2)	0.08432 (18)	0.0369 (7)	
C8	0.7274 (2)	0.8172 (3)	0.14761 (17)	0.0386 (7)	
C22	0.7307 (3)	0.8700 (3)	0.23534 (18)	0.0425 (7)	
C23	0.8062 (3)	0.8363 (3)	0.30966 (19)	0.0466 (8)	
H23	0.8569	0.7774	0.3082	0.056*	
C24	0.8016 (4)	0.8933 (4)	0.3830 (2)	0.0775 (13)	
H24	0.8507	0.8724	0.4319	0.093*	
C25	0.7296 (4)	0.9778 (4)	0.3875 (3)	0.0819 (14)	
H25	0.7297	1.0143	0.4388	0.098*	
C26	0.6569 (4)	1.0107 (4)	0.3185 (3)	0.0730 (12)	
H26	0.6065	1.0689	0.3225	0.088*	
C27	0.6573 (3)	0.9589 (3)	0.2428 (2)	0.0556 (9)	
H27	0.6076	0.9831	0.1953	0.067*	
C15	0.8246 (3)	1.0013 (3)	0.11778 (19)	0.0451 (8)	
C16	0.8442 (4)	1.2004 (4)	0.1385 (3)	0.0863 (14)	
H16A	0.8015	1.2542	0.1657	0.104*	
H16B	0.9087	1.1771	0.1796	0.104*	
C17	0.8813 (6)	1.2539 (5)	0.0680 (4)	0.124 (2)	
H17A	0.9295	1.2031	0.0445	0.185*	
H17B	0.9215	1.3219	0.0866	0.185*	
H17C	0.8175	1.2723	0.0256	0.185*	
C11	0.7228 (2)	0.9919 (3)	−0.05704 (19)	0.0409 (7)	
S1'	0.8516 (4)	0.9932 (5)	−0.0827 (3)	0.0552 (9)	0.472 (4)
C12'	0.6539 (15)	1.0804 (16)	−0.0903 (16)	0.074 (5)	0.472 (4)
H12'	0.5821	1.0924	−0.0791	0.089*	0.472 (4)
C13'	0.7087 (15)	1.154 (2)	−0.1462 (17)	0.069 (4)	0.472 (4)
H13'	0.6783	1.2169	−0.1766	0.083*	0.472 (4)
C14'	0.8130 (17)	1.1091 (17)	−0.1435 (15)	0.055 (3)	0.472 (4)
H14'	0.8625	1.1419	−0.1746	0.066*	0.472 (4)
S1	0.6308 (4)	1.0925 (5)	−0.1018 (4)	0.0706 (11)	0.528 (4)
C12	0.8224 (10)	1.0023 (18)	−0.0856 (12)	0.073 (5)	0.528 (4)
H12	0.8809	0.9514	−0.0697	0.087*	0.528 (4)
C13	0.8323 (16)	1.0985 (17)	−0.1430 (16)	0.062 (3)	0.528 (4)
H13	0.8940	1.1204	−0.1662	0.075*	0.528 (4)
C14	0.7295 (12)	1.1467 (19)	−0.1535 (15)	0.060 (3)	0.528 (4)
H14	0.7130	1.2091	−0.1888	0.072*	0.528 (4)
C18	0.8130 (3)	0.7232 (3)	0.14620 (19)	0.0449 (8)	
S2	0.8057 (3)	0.5957 (2)	0.1928 (2)	0.0789 (10)	0.632 (5)
C19	0.9075 (11)	0.7382 (13)	0.1097 (12)	0.065 (4)	0.632 (5)
H19	0.9268	0.8032	0.0829	0.078*	0.632 (5)
C20	0.9724 (10)	0.6299 (8)	0.1219 (8)	0.068 (2)	0.632 (5)
H20	1.0376	0.6191	0.1000	0.081*	0.632 (5)
C21	0.9295 (7)	0.5467 (11)	0.1678 (7)	0.077 (2)	0.632 (5)
H21	0.9619	0.4765	0.1826	0.093*	0.632 (5)
S2'	0.9239 (6)	0.7245 (7)	0.0999 (7)	0.083 (2)	0.368 (5)
C19'	0.795 (2)	0.6210 (14)	0.1856 (19)	0.111 (7)	0.368 (5)
H19'	0.7361	0.6020	0.2132	0.133*	0.368 (5)
C20'	0.8928 (18)	0.550 (2)	0.1728 (18)	0.099 (5)	0.368 (5)
H20'	0.9004	0.4766	0.1951	0.119*	0.368 (5)
C21'	0.973 (2)	0.5912 (13)	0.1278 (19)	0.099 (6)	0.368 (5)
H21'	1.0365	0.5554	0.1159	0.119*	0.368 (5)
O1	0.5657 (2)	0.7135 (2)	0.17142 (14)	0.0628 (7)	
O2	0.9203 (2)	0.9858 (2)	0.14859 (17)	0.0693 (7)	
O3	0.7748 (2)	1.1019 (2)	0.11081 (16)	0.0624 (7)	

### (III) Ethyl 4-oxo-3-phenyl-1,3-bis(thiophen-2-yl)-3,4-dihydronaphthalene-2-carboxylate Atomic displacement parameters (Å^2^)

**Table:**

	*U*^11^	*U*^22^	*U*^33^	*U*^12^	*U*^13^	*U*^23^
C7	0.0479 (18)	0.0445 (18)	0.0371 (17)	−0.0016 (15)	0.0082 (14)	−0.0013 (14)
C6	0.0351 (16)	0.0466 (18)	0.0378 (17)	0.0002 (14)	0.0087 (13)	−0.0070 (14)
C5	0.0491 (19)	0.057 (2)	0.051 (2)	−0.0095 (17)	0.0123 (15)	−0.0075 (17)
C4	0.051 (2)	0.069 (2)	0.058 (2)	−0.0145 (18)	0.0020 (17)	−0.0168 (19)
C3	0.055 (2)	0.073 (3)	0.042 (2)	−0.0038 (19)	−0.0012 (16)	−0.0138 (18)
C2	0.0466 (18)	0.059 (2)	0.0382 (18)	0.0012 (16)	0.0058 (14)	−0.0005 (16)
C1	0.0297 (14)	0.0450 (18)	0.0358 (16)	0.0059 (13)	0.0042 (12)	−0.0034 (14)
C10	0.0298 (14)	0.0426 (17)	0.0340 (16)	0.0048 (13)	0.0068 (12)	−0.0004 (13)
C9	0.0313 (15)	0.0416 (17)	0.0379 (17)	0.0010 (13)	0.0064 (12)	−0.0004 (13)
C8	0.0386 (16)	0.0447 (18)	0.0315 (16)	−0.0007 (14)	0.0039 (12)	−0.0026 (13)
C22	0.0429 (17)	0.0500 (19)	0.0358 (17)	−0.0068 (15)	0.0100 (13)	−0.0039 (14)
C23	0.0436 (18)	0.060 (2)	0.0358 (18)	−0.0077 (16)	0.0056 (14)	−0.0081 (15)
C24	0.076 (3)	0.108 (4)	0.046 (2)	−0.022 (3)	0.002 (2)	−0.005 (2)
C25	0.083 (3)	0.100 (4)	0.067 (3)	−0.024 (3)	0.027 (3)	−0.033 (3)
C26	0.081 (3)	0.066 (3)	0.081 (3)	−0.008 (2)	0.037 (3)	−0.019 (2)
C27	0.058 (2)	0.062 (2)	0.049 (2)	0.0018 (18)	0.0173 (16)	−0.0053 (18)
C15	0.0445 (19)	0.053 (2)	0.0372 (17)	−0.0040 (16)	0.0067 (14)	0.0016 (15)
C16	0.103 (3)	0.060 (3)	0.089 (3)	−0.021 (2)	−0.003 (3)	−0.018 (2)
C17	0.173 (6)	0.097 (4)	0.107 (4)	−0.066 (4)	0.041 (4)	−0.014 (3)
C11	0.0370 (16)	0.0463 (18)	0.0384 (17)	0.0006 (14)	0.0041 (13)	0.0021 (14)
S1'	0.0554 (17)	0.070 (2)	0.0429 (15)	−0.0061 (15)	0.0166 (12)	0.0080 (13)
C12'	0.079 (10)	0.073 (9)	0.065 (8)	−0.008 (7)	0.003 (7)	0.024 (6)
C13'	0.080 (8)	0.066 (7)	0.057 (6)	−0.008 (6)	−0.004 (6)	0.014 (6)
C14'	0.064 (7)	0.067 (6)	0.033 (5)	−0.017 (5)	0.010 (5)	0.013 (5)
S1	0.0579 (16)	0.0710 (19)	0.080 (3)	0.0160 (13)	0.0031 (15)	0.0267 (15)
C12	0.069 (8)	0.066 (6)	0.079 (8)	−0.004 (6)	0.004 (6)	0.011 (5)
C13	0.054 (5)	0.066 (6)	0.066 (6)	−0.002 (4)	0.006 (5)	0.004 (5)
C14	0.051 (5)	0.064 (5)	0.063 (6)	0.005 (4)	0.003 (4)	0.016 (5)
C18	0.051 (2)	0.048 (2)	0.0341 (17)	0.0095 (15)	0.0014 (14)	−0.0020 (14)
S2	0.103 (2)	0.0537 (14)	0.0785 (15)	0.0134 (14)	0.0121 (13)	0.0161 (12)
C19	0.068 (6)	0.057 (5)	0.069 (6)	0.048 (4)	0.005 (5)	−0.010 (4)
C20	0.059 (4)	0.065 (5)	0.074 (5)	0.042 (4)	0.001 (3)	−0.019 (4)
C21	0.090 (6)	0.059 (5)	0.070 (5)	0.039 (5)	−0.022 (5)	−0.009 (4)
S2'	0.064 (3)	0.083 (4)	0.108 (4)	0.010 (3)	0.029 (3)	−0.027 (3)
C19'	0.114 (11)	0.091 (11)	0.126 (13)	0.074 (9)	0.018 (9)	−0.014 (9)
C20'	0.106 (10)	0.074 (7)	0.113 (10)	0.073 (8)	0.006 (8)	−0.008 (7)
C21'	0.102 (10)	0.084 (10)	0.107 (11)	0.061 (9)	0.005 (8)	−0.020 (9)
O1	0.0707 (16)	0.0789 (18)	0.0417 (14)	−0.0247 (14)	0.0178 (12)	0.0002 (12)
O2	0.0445 (14)	0.0748 (18)	0.0813 (18)	−0.0079 (13)	−0.0092 (13)	−0.0116 (14)
O3	0.0657 (16)	0.0459 (14)	0.0711 (17)	−0.0045 (12)	0.0003 (12)	−0.0055 (12)

### (III) Ethyl 4-oxo-3-phenyl-1,3-bis(thiophen-2-yl)-3,4-dihydronaphthalene-2-carboxylate Geometric parameters (Å, º)

**Table:**

C7—O1	1.212 (4)	C16—H16B	0.9700
C7—C6	1.473 (4)	C17—H17A	0.9600
C7—C8	1.544 (4)	C17—H17B	0.9600
C6—C5	1.390 (4)	C17—H17C	0.9600
C6—C1	1.397 (4)	C11—C12	1.371 (9)
C5—C4	1.377 (5)	C11—C12'	1.384 (9)
C5—H5	0.9300	C11—S1'	1.686 (4)
C4—C3	1.375 (5)	C11—S1	1.699 (4)
C4—H4	0.9300	S1'—C14'	1.698 (9)
C3—C2	1.375 (5)	C12'—C13'	1.487 (10)
C3—H3	0.9300	C12'—H12'	0.9300
C2—C1	1.395 (4)	C13'—C14'	1.365 (9)
C2—H2	0.9300	C13'—H13'	0.9300
C1—C10	1.476 (4)	C14'—H14'	0.9300
C10—C9	1.341 (4)	S1—C14	1.697 (8)
C10—C11	1.475 (4)	C12—C13	1.481 (9)
C9—C15	1.497 (4)	C12—H12	0.9300
C9—C8	1.524 (4)	C13—C14	1.353 (8)
C8—C18	1.523 (4)	C13—H13	0.9300
C8—C22	1.535 (4)	C14—H14	0.9300
C22—C27	1.395 (4)	C18—C19	1.387 (9)
C22—C23	1.427 (4)	C18—C19'	1.397 (10)
C23—C24	1.367 (5)	C18—S2'	1.648 (6)
C23—H23	0.9300	C18—S2	1.690 (4)
C24—C25	1.336 (6)	S2—C21	1.720 (7)
C24—H24	0.9300	C19—C20	1.496 (9)
C25—C26	1.346 (6)	C19—H19	0.9300
C25—H25	0.9300	C20—C21	1.383 (8)
C26—C27	1.361 (5)	C20—H20	0.9300
C26—H26	0.9300	C21—H21	0.9300
C27—H27	0.9300	S2'—C21'	1.713 (9)
C15—O2	1.192 (4)	C19'—C20'	1.500 (10)
C15—O3	1.328 (4)	C19'—H19'	0.9300
C16—C17	1.437 (6)	C20'—C21'	1.397 (10)
C16—O3	1.458 (4)	C20'—H20'	0.9300
C16—H16A	0.9700	C21'—H21'	0.9300
			
O1—C7—C6	122.3 (3)	H17A—C17—H17B	109.5
O1—C7—C8	120.5 (3)	C16—C17—H17C	109.5
C6—C7—C8	117.1 (3)	H17A—C17—H17C	109.5
C5—C6—C1	120.2 (3)	H17B—C17—H17C	109.5
C5—C6—C7	119.2 (3)	C12—C11—C12'	108.1 (12)
C1—C6—C7	120.6 (3)	C12—C11—C10	126.6 (8)
C4—C5—C6	120.4 (3)	C12'—C11—C10	125.2 (9)
C4—C5—H5	119.8	C12'—C11—S1'	114.9 (8)
C6—C5—H5	119.8	C10—C11—S1'	119.7 (3)
C3—C4—C5	119.5 (3)	C12—C11—S1	110.3 (7)
C3—C4—H4	120.2	C10—C11—S1	123.1 (3)
C5—C4—H4	120.2	S1'—C11—S1	117.2 (3)
C2—C3—C4	120.9 (3)	C11—S1'—C14'	88.6 (8)
C2—C3—H3	119.5	C11—C12'—C13'	111.7 (17)
C4—C3—H3	119.5	C11—C12'—H12'	124.1
C3—C2—C1	120.5 (3)	C13'—C12'—H12'	124.1
C3—C2—H2	119.7	C14'—C13'—C12'	105 (2)
C1—C2—H2	119.7	C14'—C13'—H13'	127.3
C2—C1—C6	118.4 (3)	C12'—C13'—H13'	127.3
C2—C1—C10	122.0 (3)	C13'—C14'—S1'	119.4 (17)
C6—C1—C10	119.5 (3)	C13'—C14'—H14'	120.3
C9—C10—C11	120.4 (3)	S1'—C14'—H14'	120.3
C9—C10—C1	120.9 (3)	C14—S1—C11	90.2 (8)
C11—C10—C1	118.7 (2)	C11—C12—C13	116.8 (15)
C10—C9—C15	121.2 (3)	C11—C12—H12	121.6
C10—C9—C8	123.0 (3)	C13—C12—H12	121.6
C15—C9—C8	115.7 (2)	C14—C13—C12	103.0 (19)
C18—C8—C9	109.6 (2)	C14—C13—H13	128.5
C18—C8—C22	113.7 (2)	C12—C13—H13	128.5
C9—C8—C22	109.8 (2)	C13—C14—S1	119.7 (16)
C18—C8—C7	102.9 (2)	C13—C14—H14	120.2
C9—C8—C7	110.9 (2)	S1—C14—H14	120.2
C22—C8—C7	109.8 (2)	C19—C18—C19'	120.7 (10)
C27—C22—C23	117.6 (3)	C19—C18—C8	122.0 (6)
C27—C22—C8	118.1 (3)	C19'—C18—C8	117.3 (9)
C23—C22—C8	124.3 (3)	C19'—C18—S2'	114.8 (9)
C24—C23—C22	117.8 (3)	C8—C18—S2'	127.8 (4)
C24—C23—H23	121.1	C19—C18—S2	114.4 (6)
C22—C23—H23	121.1	C8—C18—S2	123.5 (3)
C25—C24—C23	122.7 (4)	S2'—C18—S2	108.7 (4)
C25—C24—H24	118.7	C18—S2—C21	94.5 (5)
C23—C24—H24	118.7	C18—C19—C20	106.8 (10)
C24—C25—C26	120.7 (4)	C18—C19—H19	126.6
C24—C25—H25	119.6	C20—C19—H19	126.6
C26—C25—H25	119.6	C21—C20—C19	115.9 (11)
C25—C26—C27	120.1 (4)	C21—C20—H20	122.0
C25—C26—H26	120.0	C19—C20—H20	122.0
C27—C26—H26	120.0	C20—C21—S2	108.2 (10)
C26—C27—C22	121.2 (4)	C20—C21—H21	125.9
C26—C27—H27	119.4	S2—C21—H21	125.9
C22—C27—H27	119.4	C18—S2'—C21'	98.3 (11)
O2—C15—O3	124.6 (3)	C18—C19'—C20'	103.3 (18)
O2—C15—C9	124.1 (3)	C18—C19'—H19'	128.3
O3—C15—C9	111.3 (3)	C20'—C19'—H19'	128.3
C17—C16—O3	110.7 (4)	C21'—C20'—C19'	121 (2)
C17—C16—H16A	109.5	C21'—C20'—H20'	119.6
O3—C16—H16A	109.5	C19'—C20'—H20'	119.6
C17—C16—H16B	109.5	C20'—C21'—S2'	103 (2)
O3—C16—H16B	109.5	C20'—C21'—H21'	128.7
H16A—C16—H16B	108.1	S2'—C21'—H21'	128.7
C16—C17—H17A	109.5	C15—O3—C16	117.2 (3)
C16—C17—H17B	109.5		
			
O1—C7—C6—C5	−16.1 (5)	C1—C10—C11—S1	69.7 (5)
C8—C7—C6—C5	160.0 (3)	C12—C11—S1'—C14'	13 (9)
O1—C7—C6—C1	162.8 (3)	C12'—C11—S1'—C14'	−3.0 (18)
C8—C7—C6—C1	−21.1 (4)	C10—C11—S1'—C14'	−177.8 (10)
C1—C6—C5—C4	0.9 (5)	S1—C11—S1'—C14'	1.5 (11)
C7—C6—C5—C4	179.8 (3)	C12—C11—C12'—C13'	1 (3)
C6—C5—C4—C3	−0.5 (5)	C10—C11—C12'—C13'	177.9 (17)
C5—C4—C3—C2	−0.2 (6)	S1'—C11—C12'—C13'	3 (3)
C4—C3—C2—C1	0.4 (5)	S1—C11—C12'—C13'	−118 (16)
C3—C2—C1—C6	0.0 (4)	C11—C12'—C13'—C14'	−2 (3)
C3—C2—C1—C10	−178.5 (3)	C12'—C13'—C14'—S1'	0 (3)
C5—C6—C1—C2	−0.7 (4)	C11—S1'—C14'—C13'	2 (2)
C7—C6—C1—C2	−179.6 (3)	C12—C11—S1—C14	−1.9 (16)
C5—C6—C1—C10	177.8 (3)	C12'—C11—S1—C14	61 (14)
C7—C6—C1—C10	−1.1 (4)	C10—C11—S1—C14	178.9 (10)
C2—C1—C10—C9	−171.3 (3)	S1'—C11—S1—C14	−0.4 (11)
C6—C1—C10—C9	10.3 (4)	C12'—C11—C12—C13	−1 (3)
C2—C1—C10—C11	7.2 (4)	C10—C11—C12—C13	−177.5 (17)
C6—C1—C10—C11	−171.2 (3)	S1'—C11—C12—C13	−166 (11)
C11—C10—C9—C15	3.0 (4)	S1—C11—C12—C13	3 (3)
C1—C10—C9—C15	−178.6 (3)	C11—C12—C13—C14	−3 (3)
C11—C10—C9—C8	−174.3 (3)	C12—C13—C14—S1	2 (3)
C1—C10—C9—C8	4.2 (4)	C11—S1—C14—C13	0 (2)
C10—C9—C8—C18	88.2 (3)	C9—C8—C18—C19	15.9 (11)
C15—C9—C8—C18	−89.2 (3)	C22—C8—C18—C19	−107.4 (11)
C10—C9—C8—C22	−146.3 (3)	C7—C8—C18—C19	133.9 (11)
C15—C9—C8—C22	36.3 (3)	C9—C8—C18—C19'	−166.1 (16)
C10—C9—C8—C7	−24.7 (4)	C22—C8—C18—C19'	70.7 (16)
C15—C9—C8—C7	157.9 (3)	C7—C8—C18—C19'	−48.0 (16)
O1—C7—C8—C18	91.3 (3)	C9—C8—C18—S2'	11.2 (6)
C6—C7—C8—C18	−84.9 (3)	C22—C8—C18—S2'	−112.1 (6)
O1—C7—C8—C9	−151.7 (3)	C7—C8—C18—S2'	129.2 (6)
C6—C7—C8—C9	32.1 (4)	C9—C8—C18—S2	−167.6 (3)
O1—C7—C8—C22	−30.1 (4)	C22—C8—C18—S2	69.1 (4)
C6—C7—C8—C22	153.7 (3)	C7—C8—C18—S2	−49.6 (3)
C18—C8—C22—C27	178.5 (3)	C19—C18—S2—C21	−0.5 (11)
C9—C8—C22—C27	55.3 (3)	C19'—C18—S2—C21	170 (14)
C7—C8—C22—C27	−66.9 (4)	C8—C18—S2—C21	−177.2 (5)
C18—C8—C22—C23	0.1 (4)	S2'—C18—S2—C21	3.8 (6)
C9—C8—C22—C23	−123.0 (3)	C19'—C18—C19—C20	1 (2)
C7—C8—C22—C23	114.8 (3)	C8—C18—C19—C20	178.8 (8)
C27—C22—C23—C24	−0.1 (5)	S2'—C18—C19—C20	−33 (8)
C8—C22—C23—C24	178.2 (3)	S2—C18—C19—C20	2.0 (16)
C22—C23—C24—C25	0.3 (6)	C18—C19—C20—C21	−3.2 (19)
C23—C24—C25—C26	0.2 (7)	C19—C20—C21—S2	2.9 (15)
C24—C25—C26—C27	−0.9 (7)	C18—S2—C21—C20	−1.4 (9)
C25—C26—C27—C22	1.1 (6)	C19—C18—S2'—C21'	145 (10)
C23—C22—C27—C26	−0.6 (5)	C19'—C18—S2'—C21'	−3 (2)
C8—C22—C27—C26	−179.0 (3)	C8—C18—S2'—C21'	−179.9 (11)
C10—C9—C15—O2	−119.3 (4)	S2—C18—S2'—C21'	−1.0 (12)
C8—C9—C15—O2	58.2 (4)	C19—C18—C19'—C20'	−2 (3)
C10—C9—C15—O3	62.5 (4)	C8—C18—C19'—C20'	−179.8 (15)
C8—C9—C15—O3	−120.1 (3)	S2'—C18—C19'—C20'	3 (3)
C9—C10—C11—C12	69.2 (13)	S2—C18—C19'—C20'	−12 (12)
C1—C10—C11—C12	−109.3 (12)	C18—C19'—C20'—C21'	−1 (4)
C9—C10—C11—C12'	−106.8 (15)	C19'—C20'—C21'—S2'	0 (4)
C1—C10—C11—C12'	74.7 (15)	C18—S2'—C21'—C20'	2 (2)
C9—C10—C11—S1'	67.6 (4)	O2—C15—O3—C16	3.7 (5)
C1—C10—C11—S1'	−111.0 (4)	C9—C15—O3—C16	−178.1 (3)
C9—C10—C11—S1	−111.7 (4)	C17—C16—O3—C15	96.6 (5)
